# Mosquito pornoscopy: Observation and interruption of *Aedes aegypti* copulation to determine female polyandric event and mixed progeny

**DOI:** 10.1371/journal.pone.0193164

**Published:** 2018-03-08

**Authors:** Danilo O. Carvalho, Samira Chuffi, Rafaella S. Ioshino, Isabel C. S. Marques, Regina Fini, Maria Karina Costa, Helena R. C. Araújo, André L. Costa-da-Silva, Bianca Burini Kojin, Margareth L. Capurro

**Affiliations:** 1 Departamento de Parasitologia, Instituto de Ciências Biomédicas, Universidade de São Paulo, São Paulo, SP, Brazil; 2 Instituto Nacional de Ciência e Tecnologia em Entomologia Molecular, Rio de Janeiro, RJ, Brazil; Centro de Pesquisas René Rachou, BRAZIL

## Abstract

Ades aegypti is the most important arbovirus vector in the world, and new strategies are under evaluation. Biological studies mentioning the occurrence of a second mate in *Aedes aegypti* can interfere with vector control program planning, which involves male mosquito release technique. This study presents different experiments to show the occurrence of mixed progeny. Mixed male crosses (using a combination of different type of males in confinement with virgin females) showed no polyandric female. Individual crosses with male substitution in every gonotrophic cycle also did not show any polyandric female. Individual crosses with a 20 minutes interval, with subsequent male change, showed that only a few females presented mixed offspring. The copulation breach in three different moments, group A with full coitus length, group B the coitus was interrupted in 5–7 seconds after the start; and group C, which the copulation was interrupted 3 seconds after started. In summary, group A showed a majority of unique progeny from the first male; group B showed the higher frequency of mixed offspring and group C with the majority of the crosses belonging to the second male. To conclude, the occurrence of a viable second mate and mixed offspring is only possible when the copulation is interrupted; otherwise, the first mate is responsible for mixed progeny.

## Introduction

The mosquito *Aedes aegypti* is one of the most relevant disease vectors in the world due to its capacity to adapt to the urban environment and being able to transmit different pathogens, such as Dengue, Chikungunya and Zika virus [1–3]. There are new attempts at the control of *Ae*. *aegypti* population using *Wolbachia-*infected mosquitoes, Genetically Modified Mosquitoes (GMM) and sterile mosquitoes using ionizing radiation (so-called Sterile Insect Technique–SIT) [4]. They are all technologies under evaluation in different countries, and their primary target is to suppress mosquito population and decrease (and eliminate) arboviruses transmission [4,5] using male releases. The aim is to provide a mating competitivity disadvantage for the wild-type male and increase the number of successfully mated females with the desired released male. Besides operational aspects of those technologies, additional ecological and biological factors need to be clarified and not only to guarantee safeness to the human population but also to understand the behavior of the technology under different circumstances [6–8]. One crucial factor is to determine the mechanisms which females of this species become polyandric, or can mate with several males.

Mosquito mating process (coitus) consists of three phases, the coupling (from recognition to genital contact), copulation (engagement of the genitalia and initiation of semen transfer) and insemination (deposition of spermatozoa and additional secretion into the bursa and later migration to the spermatheca). *Ae*. *aegypti* (males and females) after adult emergence, they need 36 to 48 hours to be fully able to mate, during this period the male genitalia turns 180° to make copulation process possible [9,10].

Studies on mosquito mating behaviors are difficult to perform due to the sophisticated constituency (swarm formation) and its uneven spatial distribution [11]. These factors also include behavior parameters, such as recognition, wing beats and pheromones [9–11]. Yuval et al. (2006) mention that some *Aedes* species may swarm near the host, and because they are continuous breeders in small containers of low volume they highly disperse at low densities [12]. In *Ae*. *aegypti*, the copulation and insemination usually take around six to ten seconds, and this period is sufficient to transfer enough amount of sperm for the entire female’s lifespan [13,14]. In laboratory conditions, copulation may take longer, as observed by Oliva and colleagues (2013) for *Ae*. *albopictus*, which could take up to 45 seconds compared to 8 seconds in natural conditions [15]. The study also mentions that when copulation occurs in a small confinement space, polyandry happens more often [16].

The additional material transfer during copulation has a significant role on female behavior, such as stimulating the female to find a blood meal, and critical from vector control point of view, the female becomes refractory to subsequent (future) mating. The male accessory gland produces the seminal fluid which contains many proteins and peptides which are involved in modulating the female responses to host-seek, mating refractoriness, stiffening of the spermatheca, among others. So, the mating is not only for egg production, but it also has an essential factor in mosquito behavior [16–18].

Even after storing enough sperm for their lifespan, *Aedes* mosquito females can mate with other subsequent males, but usually, the offspring belongs to the first male [10,19]. However, some polyandrous females and females which their offspring belongs to different males were found among mosquitoes collected from the field [20–23]. This information is crucial to new vector control strategies mentioned previously since they require releasing millions of male mosquitoes. Moreover, due to polyandry, the techniques would require significant changes to a sustained release protocol regarding the frequency of males to be produced, its frequency to be released and the necessary number of releases in the field to compensate and guarantee the success of all techniques [24–26].

Although some studies have demonstrated the occurrence of a re-mating event, even in low frequencies, they do not explore the mechanism by how this phenomenon occurs. Once mated females are capable of multiple mating, they can empty the bursa or use the sperm for the next progeny, and this mechanism is not yet determined. The described experiments in this paper considered the study of Degner and Harrington (2016) [27], where the majority of polyandric females occurred in 0–2 hours post-mating. Thus, we focused on determining a fully capable mixed-offspring mother as a polyandric female, and the moment a female could be able to have a mixed progeny.

## Material and methods

### Mosquito rearing and manipulation

The *Aedes aegypti* strains were reared using standard laboratory procedures [28,29]. Briefly, eggs were hatched in previously boiled water jars for around 12 hours and L1 larvae placed in plastic trays with 30x20x10 cm with 2 L distilled water with 300 larvae/tray. Larvae were fed with finely grounded fish food (Sera Vipan Premium, Germany) throughout all instars. Pupae were sorted using a glass plate sorter, and each gender was kept separated until adult emergence. Adults were fed with 10% sucrose solution *ad libitum* using cotton wool balls. Pre-mated female mosquitoes were blood fed on anesthetized mice when necessary. For female blood feeding the University of Sao Paulo Ethics Committee approved the method under the certificate number:186, 187 and 188/12/CEUA.

For all experiments using individual female egg collection, pregnant/blood-fed females were transferred to flat-bottom glass tube (2.5 Ø x 10 cm) containing a wet cotton ball covered by a wet circular filter paper on the bottom and the tube closed with a dry cotton ball. The insectary rearing conditions were 27°C (±1°C), 80% humidity (±10%), and 16/08-hour light/dark photoperiod with 4 hours dusk/dawn.

For all experiments, the Higgs strain was used as wild-type, and the respective transgenic line N2, described by [30] to quickly identify the offspring. The N2 transgenic line express eGFP in the eyes driven by the 3xP3 promoter as a dominant characteristic, all offspring resulted from an N2 male, and a wild-type female is heterozygous and has the fluorescent eyes when submitted under fluorescent light.

### Progeny type determination—Fluorescence screening

In all experiments, progeny type was determined under the fluorescent light as unique or mixed progeny. Unique progeny was defined when the larval fluorescent status was positive, or all larvae were negative. Mixed progeny was defined when the larval fluorescent status was both positive and negative. Progeny of each cross was submitted as larvae (L3-L4) under fluorescent light in excitation 480/40 nm, extinction 510 nm in Leica MZ FLIII stereomicroscope (Leica Microsystem, Heerbrugg, Switzerland).

### Mixed male cross

Pupae were sorted, and males were kept separated from females to avoid coitus. Copulation occurred in plastic containers of 08 Ø x 10 cm (mating container) with ten adult virgin males of both lines (five wild-type and five transgenic—with at least three days after emergence) and ten adult virgin wild-type females allowed to mate for 24 hours. Females blood fed, and three days after blood feeding they (individually) had access to an oviposition site–females which did not blood feed were substituted). After the first gonotrophic cycle, the collected eggs dried in lab conditions and the number of eggs, the number of larvae and the hatch rate were recorded. This process was repeated for the following three subsequent gonotrophic cycles (total of four cycles). The progeny type was determined under fluorescent light, as described previously, as unique progeny (the total isofemale offspring belongs to one male type only) or mixed (the total isofemale offspring belongs to two male type). The results combine three replicates with 30 crosses in total.

### Consecutive male exchange

**Fig 1:**
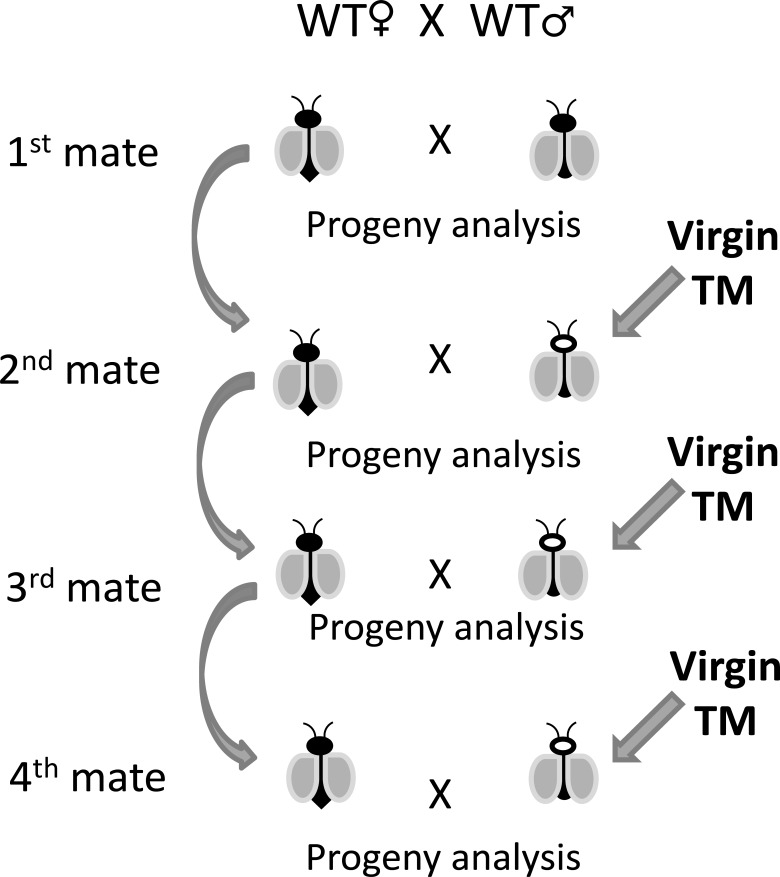
Method illustration of the male exchange experiment.

Wild-type female and male pupae (wild-type and transgenic) were isolated after sorting to avoid coitus, and they remained isolated for up to 3 days after emergence. There were two experimental groups, the first group (group A), it was constituted by 30 individual couples of one wild-type female and one wild-type male, they mated for 24 hours, and the male was removed from the cage. All females were transferred to a lab cage; they received a blood meal, and eggs were collected/counted. Later a transgenic male was placed in the cage and remained for another 24 hours. This substitution with a new and virgin transgenic male happened before every subsequent gonotrophic cycle of the same wild-type female (Fig 1). In the group B, the order of the first male was inverted, starting with a virgin transgenic male and substitute by a virgin wild-type male for the subsequent cycles. The data collection happened for six gonotrophic cycles in each group, and the offspring paternity evaluation determined as previously described. This experiment was performed in three replicates.

### 20 minutes mating interval

**Fig 2:**
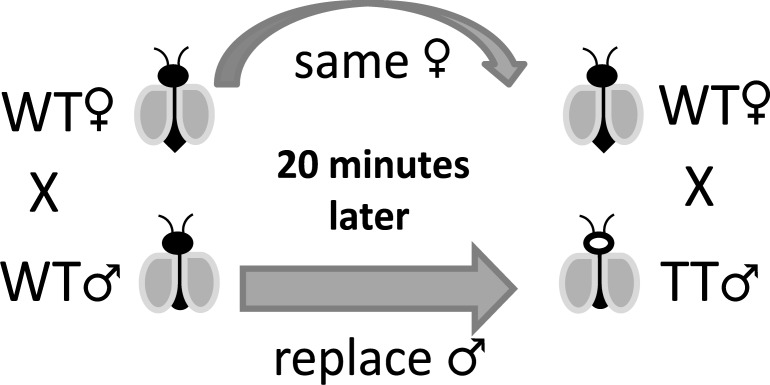
Method illustration of the 20 minutes interval experiment.

A total of 10 individual couples (wild-type adults with up to 3 days after emergence) were placed in plastic containers and stayed for 20 minutes to perform mating. After this period the wild-type male was substituted by a transgenic male, and the couple stayed for at least 24 hours before the transgenic male being also removed (Fig 2). Like previous experiments the inverted cross was also performed, starting with a transgenic male and then substituting by a wild-type one. All females had access to a blood meal, and they performed individual oviposition. The number of eggs and its hatch rate were recorded, and the progeny status was evaluated as previously. This experiment was performed in three replicates.

### Interrupted coitus

**Fig 3:**
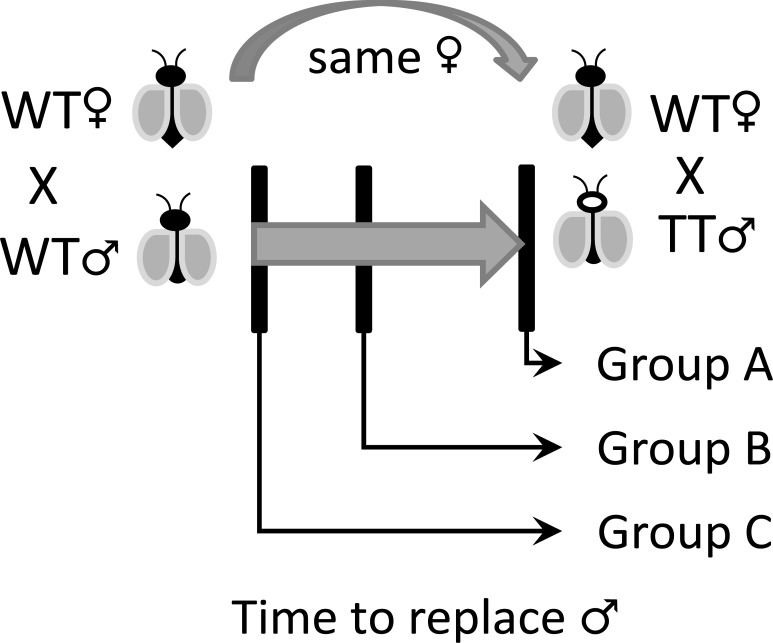
Method illustration of the interrupted coitus experiment.

Three mating groups (with at least 30 crosses using adults with three days after emergence) were defined according to the total amount of time to perform copulation, group A—the couple performed the coitus without interruption (full-time length). Group B—the couple was interrupted in the middle of coitus (5–7 seconds), and group C—couple was interrupted as soon as the male has to grab females’ genitalia (Fig 3). The observer interrupted the coitus by a strong air blow inside the mating container using the mouth aspirator by the time limit of each group. The transgenic males immediately replaced the wild-type male; the new couple remained together for the next 24 hours. After this period, males were removed, and females were blood fed and had their number of eggs and hatch rate recorded individually. The paternity was determined as above. The crosses which could not fit in any appropriate mating group were discarded. This experiment was performed in three replicates.

### Statistical analysis

All statistical analyses were performed by scripts written in R language implemented in the RStudio software [31,32].The statistical analyses for all experiments with a continuous type of data (number of eggs per female and hatch rate) were submitted for a normality test (Shapiro-Wilk Normality test [33]) considering nonparametric data with a p-value higher than 0.05; analyses employed the core statistical package: stats. The plot of histograms and normal distribution curves, linear models/general linear models, ANOVA and graphical plots of each set of data of the continuous data were done using the following R packages: rcompanion, ggplot2, multcompView, plyr, knitr. In all cases, p was considered significant when lower than 0.05. An ordered logistic regression model was used to estimate the probability of crosses in different categories. This specific model and its respective plot used the R packages: MASS, reshape2, and ggplot2. Data bank is available in S1 File.

## Results

### Mixed male cross

In total, 30 crosses with 30 virgin females and 30 virgin males (15 wild-types and 15 transgenics) performed copulation in lab containers. Table 1 shows the mean number of eggs and its mean hatch rate for each gonotrophic cycle (statistical analysis is provided in S2 File). The number of crosses (isofemale laying eggs) was counted, and the frequency of crosses with the mixed or the unique type of progeny is also shown in Table 1. In this experiment, all crosses (100%) only had unique offspring type, meaning no polyandric event was observed in aggregate mating condition.

**Table 1:** Average number of eggs, mean hatch rate and number of crosses with mixed/unique type of progeny in mixed male crosses.

Gonotrophic cycle	# eggs/ female*	Hatch rate (%)*	Mixed progeny	Unique progeny
1^st^	137.13 (21.9)	91.2 (10.9)	0 (0%)	30 (100%)
2^nd^	128.37 (13.8)	88.4 (7.2)	0 (0%)	30 (100%)
3^rd^	114.03 (34.7)	85.6 (8.9)	0 (0%)	30 (100%)
4^th^	124.53 (18.0)	86.0 (6.9)	0 (0%)	30 (100%)

*—The values between brackets represent the standard deviation.

### Consecutive male exchange

In groups A and B (Table 2), the first gonotrophic cycle had 100% of unique progeny from the first male, as expected. From the subsequent gonotrophic cycles (from 2^nd^ to 6^th^) no cross showed mixed progeny, or polyandric event, remaining 100% of crosses with unique progeny despite the virgin male change. There was no difference regarding the crosses between group A and B. Statistical analysis regarding the number of eggs and hatch rate are available in the S2 File.

**Table 2:** Average number of eggs and hatch rate through a consecutive male change throughout different gonotrophic cycles and its progeny status.

Male type	Gonotrophic Cycle	# Eggs/ female*	Hatch rate (%)*	Mixed progeny	Unique progeny
Wild-type	1^st^	123.8 (13.5)	84.6 (17.9)	-	30 (100%)
Transgenic (group A)	2^nd^	128.2 (12.8)	64.3 (18.2)	0 (0%)	30 (100%)
	3^rd^	101.9 (26.4)	91.0 (8.8)	0 (0%)	30 (100%)
	4^th^	92.3 (36.7)	86.5 (11.1)	0 (0%)	30 (100%)
	5^th^	100.7 (45.7)	78.1 (21.4)	0 (0%)	30 (100%)
	6^th^	133.9 (31.1)	73.3 (25.9)	0 (0%)	30 (100%)
Transgenic	1^st^	129.4 (34.8)	79.0 (15.0)	-	30 (100%)
Wild-type (group B)	2^nd^	136.9 (38.5)	69.2 (15.9)	0 (0%)	30 (100%)
	3^rd^	116.4 (47.1)	86.6 (11.5)	0 (0%)	30 (100%)
	4^th^	146.5 (17.1)	70.9 (20.6)	0 (0%)	30 (100%)
	5^th^	130.6 (28.7)	76.1 (24.7)	0 (0%)	30 (100%)
	6^th^	145.3 (27.4)	76.5 (14.8)	0 (0%)	30 (100%)

*—The values between brackets represent the standard deviation.

The first gonotrophic cycle occurred with a virgin female mating with one of the males (group A wild-type and group B transgenic), they were replaced by a different type of male (group A transgenic and group B wild-type) every subsequent gonotrophic cycle (up to the 6^th^).

### 20 minutes mating interval

The interval mating had a total of 30 crosses individually observed and followed by two gonotrophic cycles. The number of eggs per female and its hatch rate statistical analysis are available in the S2 File. Table 3 shows the number of crosses (and frequencies) on each gonotrophic cycle, which only four presented mixed progeny and 26 unique progeny type. Considering these number, the percentage of polyandric females was 13.3%. The number of crosses with mixed progeny on the first and second gonotrophic cycle is the same (total of 4 crosses), this might suggest that they are the same females on both gonotrophic cycles.

**Table 3:** Average number of eggs, mean hatch rate and number of crosses with mixed/unique type of progeny in 20 minutes interval for first male cross.

Gonotrophic cycle	# eggs/ female *	Hatch rate (%) *	Mixed progeny	Unique progeny
1^st^	117.0 (26.8)	79.8 (14.9)	4 (13.3%)	26 (86.7%)
2^nd^	125.7 (32.4)	82.9 (13.7)	4 (13.3%)	26 (86.7%)

*—The values between brackets represent the standard deviation.

### Interrupted coitus

A total of 227 individual couples were observed for each mating group, previously determined (120 crosses with a transgenic male as the first male and 107 crosses with a wild-type male). For the number of eggs per female and its hatch rate, the statistical analysis details and average data are provided in the S2 File. The data was subset into two groups, one using a transgenic male (TM) as first male and a wild-type male (WM) as the second, and the second subset in an inverted order (Table 4). The first subset for group A (no coitus interruption) had 100% of the crosses from the first male (TM), 0% from males 1 and 2 (TM/WM) and 0% from the second male (WM). For the mating group B (interruption between 5–7 seconds after coitus initiation), 51.4% of the crosses belong to the first male (TM), while 40.0% belongs to both males (TM/WM) and 8.6% belongs to the second male (WM). The mating group C (interruption between 1–3 seconds after coitus initiation) had 2.3% of the crosses from the first male (TM), 25.0% from both males (TM/WM) and 72.7% from the second male (WM).

**Table 4:** Number of crosses of each interrupted mating group according to the initial male.

1st mating male	Progeny type	Mating group					
		A		B		C	
**Transgenic**	Male 1	41	100%	18	51.4%	1	2.3%
	Male 1&2	0	0.0%	14	40.0%	11	25.0%
	Male 2	0	0.0%	3	8.6%	32	72.7%
**Wild-type**	Male 1	37	97.4%	15	42.9%	1	2.9%
	Male 1&2	1	2.6%	16	45.7%	5	14.7%
	Male 2	0	0.0%	4	11.4%	28	82.4%

For the second subset (Table 4), the mating group A had 97.4% from the first male (WM), 2.6% from both males (WM/TM) and no cross produced progeny from the second male. The mating group B had 42.9% of the crosses from the first male (WM), 45.7% of both males (WM/TM) and 11.4% from the second male (TM). The final mating group C had 2.9% of the crosses from the first male (WM), 14.7% from both males (WM/TM) and 82.4% from the second male (TM).

**Fig 4:**
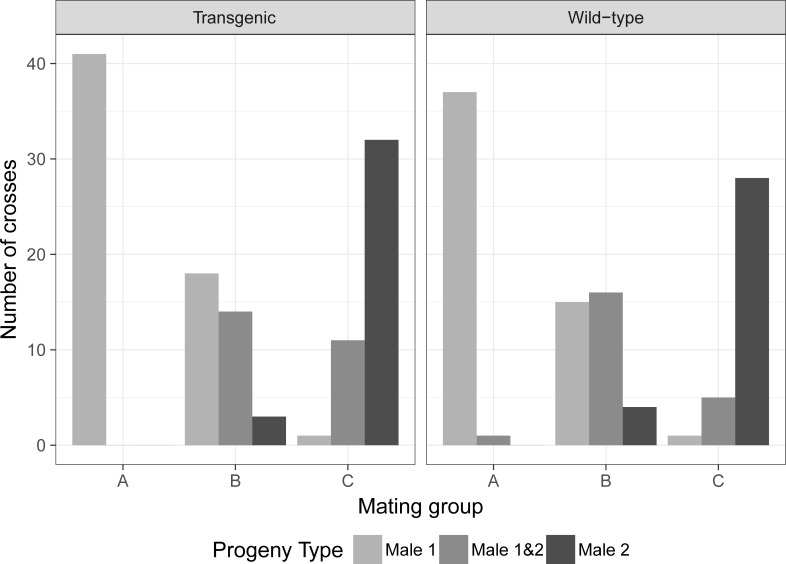
Total number of crosses among the progeny type and the initial male.

In both subsets, the group A presented the highest number of crosses which the progeny type is identical to the first male that had contact with the female with 100 and 97.4% of the crosses. Group B shows the highest concentration of polyandric crosses with 40 and 45.7% of them. Group C had the highest number of crosses which the progeny type belongs predominantly to the second male with 72.7 and 82.4% of the crosses in each dataset (Fig 4).

**Fig 5:**
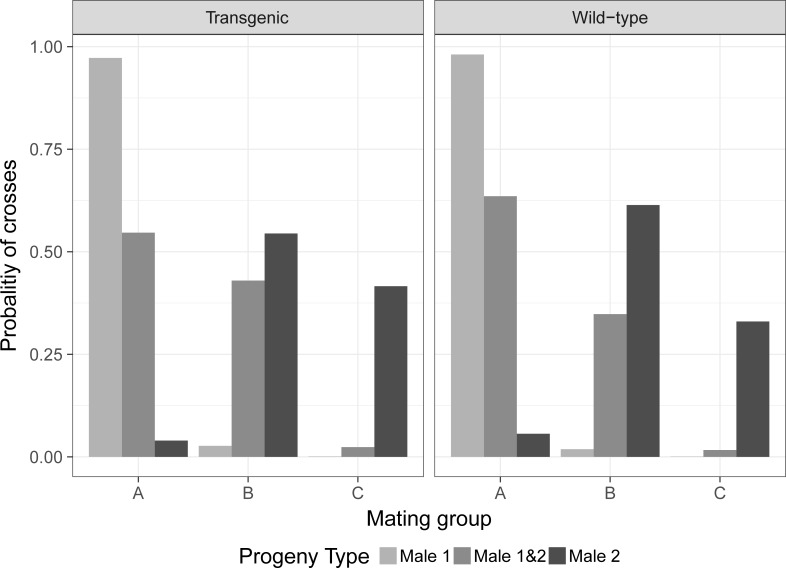
Model probability distribution of crosses among the progeny type and the initial male.

An ordered logistic regression model was performed using the mating group in function of the progeny type (male 1, male 1&2 and male 2) and the first mating male (transgenic and wild-type). The regression model had a coefficient for progeny type of 3.373 (with a standard error—SE of 0.3434 and t-value 9.821) and male type of -0.369 (SE 0.315 and t-value of -1.172. The Residual Deviance was equal to 271.51 and the AIC equal to 279.51. The code used for the analysis is available in S2 File. Fig 5 shows the plot obtained from the ordered logistic regression model, with the probability of each mating group (A, B or C) to produce a polyandric event (progeny type) when the first male is transgenic or wild-type. About the type of male, the model shows that there was no statistical difference between transgenic or wild-type first male mating with a p-value equals to 0.241 (p-values table in the S2 File), while there was a p < 0.05 for all mating groups about the progeny type.

## Discussion

The study from Degner and Harrington (2016) define polyandric females as a female which received sperm from multiple males, they also mention that the majority of polyandric events occurs in 0–2 hours post-mating interval. They found sperm of the second male in females’ bursa, but they do not specify whether those females could use the second male sperm to produce mixed progeny [27]. In our work, we defined as polyandric females, the female which was able to provide an offspring from two different males (a wild-type and a transgenic).

Herein, the mixed male cross experiment resulted in no polyandric event, despite the possibility of polyandry females to occurs in laboratory cages and conditions [27,34]. The absence of a polyandric event might be related to the insect density and the size of the mating container, which could be not sufficient to promote it. However, males after many coitus can be depleted of sperm and increase the chances of a polyandric female to occur [34,35]. With this information, we could not determine in our experiments if polyandric females happened between males of the same type.

The first male mating replacement experiment showed that even after six consecutive replacements and egg collection, none of the females were able to produce a mixed progeny, this result can be compared with the previously described from Gwadz (1970) and in particular to Spielman (1967) [34,36]. It means that even if a fully-mated female had coitus with a second male, this sperm would not be stored in the spermathecas for egg production, although the second sperm can be kept for some time inside the bursa, as showed by Degner and Harrington (2016) [27].

In our study, the polyandric event was observed in a 20 minutes interval between the first and the second male, presented to the same female. From all crosses, we had a 13.4% polyandric females, which is comparable to semi-field and field collection studies [21,23,37,38]. However, it is worth to mention that during the 20 minutes interval experiment, some crosses had their copulation interrupted to replace the male. The interruption could have contributed to the polyandry.

In this sense, controlling the copulation breach (group B) increases the chances of the second male to deposit sperm in the female bursa and contributes for a mixed offspring (when compared to group A), this can be correlated with the work described by Degner and Harrington (2016) [27]. On the other hand, group C showed an inverted scenario from group A, the majority of the crosses resulted in offspring from the second male, which might be related to the amount of sperm transferred to females. If the amount is not sufficient, females can accept a second load from a different male [34,36]. Group C shows that the time to deposit sperm and guarantee a viable offspring was not enough, which enables the second male to be successful, despite female refractoriness.

The accessory gland produces the seminal fluid, which is constituted by several proteins and molecules to guarantee the sperm viability. Those proteins play a significant role in modifying female physiology and contribute to refractoriness [16,18,21,39–41]. Due to this seminal fluid, females keep laying eggs from the first-mated male even after several gonotrophic cycles and exhaust the sperm over time. The critical factor for polyandry is a 0–2 hours period which provides an experimental time-window to determine how often polyandry occurs [27]. A recent study from Duvall et al. (2017), demonstrated that the mating period for polyandry events to occur is concise and the Head Peptide-I (HP-I) and the NeuroPeptide Y-like Receptor 1 (NPYR1) both have an essential role in female refractoriness and polyandric event formation [42].

The coitus interruption is one of the key factors to increase the chances of the polyandric event to occur. This interruption could be any source of disturbance, which allows the couple to disassemble and would permit another male to take place [34]. Field conditions could also contribute to the increase of polyandric events, where the resources are limited, and developmental conditions are more drastic [43–48]. Males reared in field conditions with a higher fitness cost might transfer less sperm during coitus, and even after a full mating time, females could not be fully inseminated. The unfit male situation allows a second male to transfer an additional sperm load. Those contributing factors need to be described and evaluate its impacts on mosquito control programs that depend on copulation to achieve population suppression or substitution, similarly evaluated for other insects [5].

## Conclusion

The results suggest that in laboratory conditions, females which had an uninterrupted coitus will produce progeny from this first male in all subsequent gonotrophic cycles, even though this female still have subsequent and consecutive coitus with later and different types of males. However, polyandric events can occur more frequently when coitus is interrupted in the middle of it. The polyandric event is associated with the amount of sperm deposited by each male and the time gap between the first and the second mate. Although many information is available regarding polyandric females in laboratory conditions, the influence of those factors to promote polyandry in field conditions remain to be discussed.

## Supporting information

S1 File Data bank. Data bank containing all data used for the analysis of each experiment.
(XLSX)

S2 File Statistical analysis. Detailed statistical analysis conducted for each experiment and extra information.
(PDF)
