# Correction: Mosquito pornoscopy: Observation and interruption of *Aedes aegypti* copulation to determine female polyandric event and mixed progeny

**DOI:** 10.1371/journal.pone.0209146

**Published:** 2018-12-10

**Authors:** Danilo O. Carvalho, Samira Chuffi, Rafaella S. Ioshino, Isabel C. S. Marques, Regina Fini, Maria Karina Costa, Helena R. C. Araújo, André L. Costa-da-Silva, Bianca Burini Kojin, Margareth L. Capurro

The following information is missing from the Funding section: This study was supported by the São Paulo Research Foundation (FAPESP; Grant no. 2013/19921-9). Additionally, all authors were supported by Coordenação de Aperfeiçoamento de Pessoal de Nível Superior (CAPES).
