# Supplementary material for: Mosquito pornoscopy: Observation and interruption of Aedes aegypti copulation to determine female polyandric event and mixed progeny
Source: PLoS One. 2018 Mar 8;13(3):e0193164. doi: 10.1371/journal.pone.0193164 (PMC5843176; doi:10.1371/journal.pone.0193164)

Mosquito Pornoscopy - Statistics

Mixed Progeny

Experimental description in material and methods section.

Normality Test

```
##           n_eggs                         hatch_rate
## statistic 0.9742797                      0.8124048
## p.value   0.02116082                     4.524147e-11
## method    "Shapiro-Wilk normality test"  "Shapiro-Wilk normality test"
## data.name "X[[i]]"                       "X[[i]]"
```

The Shapiro-Wilk normality test showed that for the number of eggs per female from the mixed progeny dataset is nonparametric, as well as the hatch rate with p value 0.0211608 and 4.524146710^{-11}, respectively. The histogram of each group is presented below.

Histogram and QQ-plot

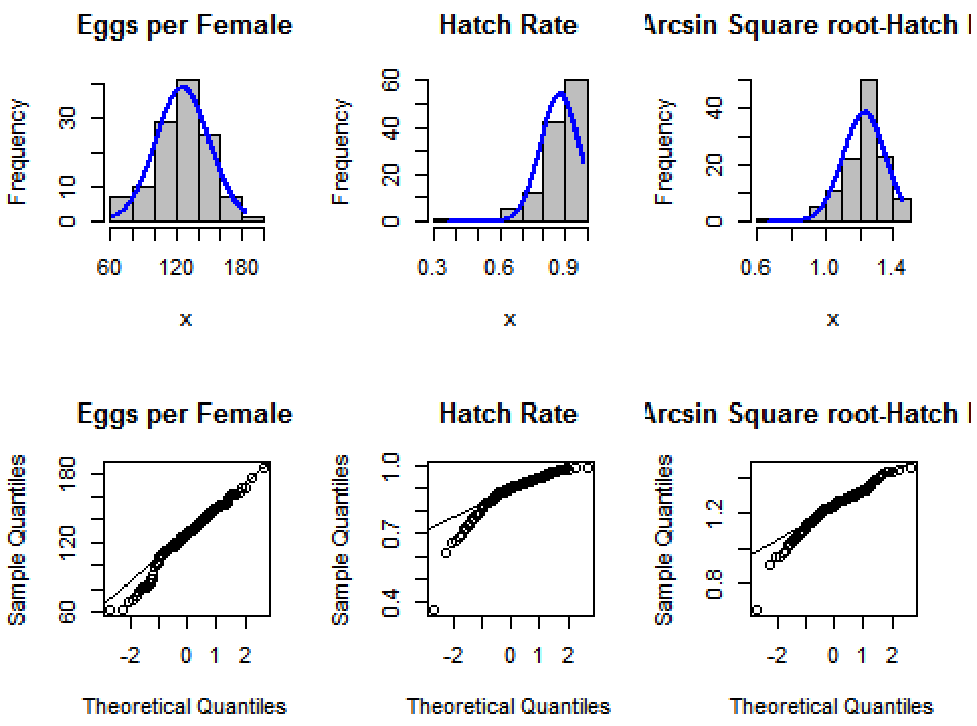

The number of eggs per female can be considered as parametric based ond histogram distribution of frequencies and the plot of sample and theoretical quantiles. However the hatch rate was considered nonparametric using the same approach. For this case an arcsin square root transformation was performed to obtain a parametric distribution.

Linear Model for the number of eggs per female

According to the plot of histogram and QQ-plot, the number of eggs per female was submited to a linear model and check among the different gonotrophic cycles if there was any significant statistical difference among them

```
## [1] "Linear Model"

##
## Call:
## lm(formula = mixed_mating$n_eggs ~ mixed_mating$gono_cycle_1)
##
## Residuals:
##     Min      1Q  Median      3Q     Max
## -52.133 -13.658  -0.583  15.567  52.967
##
## Coefficients:
##                             Estimate Std. Error t value Pr(>|t|)
## (Intercept)                  137.133      4.282  32.029  < 2e-16 ***
## mixed_mating$gono_cycle_12    -8.767      6.055  -1.448  0.15036
## mixed_mating$gono_cycle_13   -23.100      6.055  -3.815  0.00022 ***
## mixed_mating$gono_cycle_14   -12.600      6.055  -2.081  0.03964 *
## ---
## Signif. codes:  0 '***' 0.001 '**' 0.01 '*' 0.05 '.' 0.1 ' ' 1
##
## Residual standard error: 23.45 on 116 degrees of freedom
## Multiple R-squared:  0.1145, Adjusted R-squared:  0.09158
## F-statistic: 4.999 on 3 and 116 DF,  p-value: 0.002691

## [1] "ANOVA"

##                             Df Sum Sq Mean Sq F value  Pr(>F)
## mixed_mating$gono_cycle_1   3   8247  2749.0   4.999 0.00269 **
## Residuals                 116  63793   549.9
## ---
## Signif. codes:  0 '***' 0.001 '**' 0.01 '*' 0.05 '.' 0.1 ' ' 1

## [1] "TUKEY - post hoc test"

##   Tukey multiple comparisons of means
##     95% family-wise confidence level
##
## Fit: aov(formula = n_eggs_mixed_mating_lm)
##
## $`mixed_mating$gono_cycle_1`
##           diff        lwr       upr     p adj
## 2-1  -8.766667 -24.549924  7.016591 0.4723829
## 3-1 -23.100000 -38.883258 -7.316742 0.0012439
## 4-1 -12.600000 -28.383258  3.183258 0.1654538
## 3-2 -14.333333 -30.116591  1.449924 0.0892548
## 4-2  -3.833333 -19.616591 11.949924 0.9211615
## 4-3  10.500000  -5.283258 26.283258 0.3108609
```

Residuals plot of the number of eggs per female

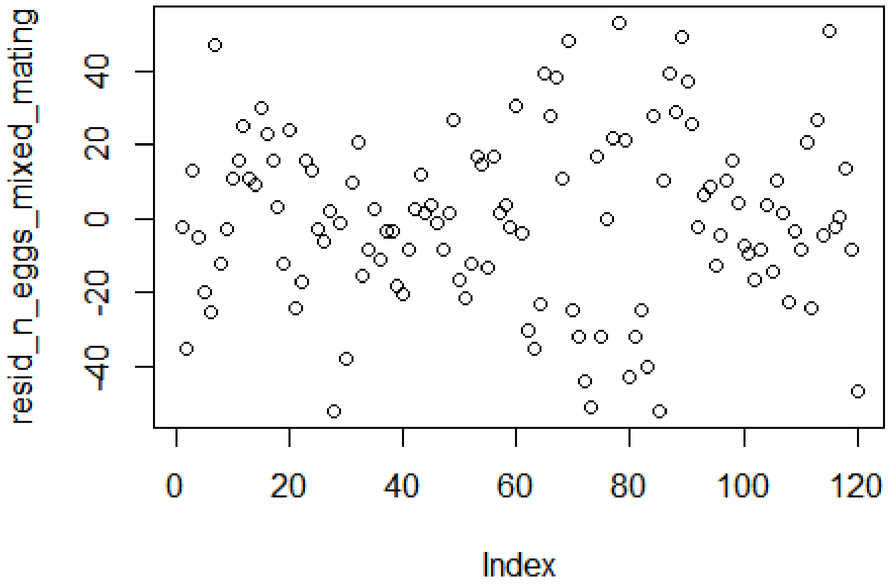

Graph number of eggs per female by gonotrophic Cycles

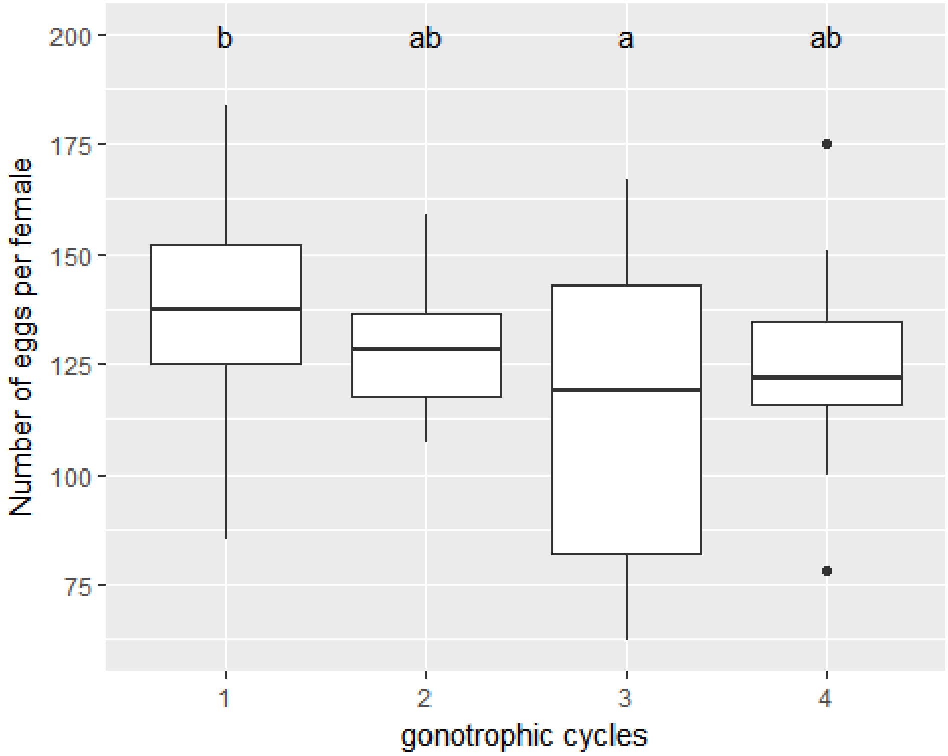

Linear Model for the transformed hatch rate

According to the plot of histogram and QQ-plot, the hatch rate was submited to a arcsin square root transformation and later to a linear model and check among the different gonotrophic cycles if there was any significant statistical difference among them.

```
## [1] "Linear Model"

##
## Call:
## lm(formula = mixed_mating$hatch_rate ~ mixed_mating$gono_cycle_1)
##
## Residuals:
##      Min       1Q   Median       3Q      Max
## -0.54694 -0.02803  0.02071  0.05351  0.12064
##
## Coefficients:
##                             Estimate Std. Error t value Pr(>|t|)
## (Intercept)                  0.91165    0.01580  57.708   <2e-16 ***
## mixed_mating$gono_cycle_12 -0.02772    0.02234  -1.241   0.2173
## mixed_mating$gono_cycle_13 -0.05591    0.02234  -2.503   0.0137 *
## mixed_mating$gono_cycle_14 -0.05189    0.02234  -2.323   0.0219 *
## ---
## Signif. codes:  0 '***' 0.001 '**' 0.01 '*' 0.05 '.' 0.1 ' ' 1
##
## Residual standard error: 0.08653 on 116 degrees of freedom
## Multiple R-squared:  0.06449,    Adjusted R-squared:  0.0403
## F-statistic: 2.666 on 3 and 116 DF,  p-value: 0.0511

## [1] "ANOVA"

##                            Df Sum Sq  Mean Sq F value Pr(>F)
## mixed_mating$gono_cycle_1   3 0.0599 0.019958   2.666 0.0511 .
## Residuals                 116 0.8685 0.007487
## ---
## Signif. codes:  0 '***' 0.001 '**' 0.01 '*' 0.05 '.' 0.1 ' ' 1

##   Tukey multiple comparisons of means
##     95% family-wise confidence level
##
## Fit: aov(formula = hatch_mixed_mating_lm)
##
## $`mixed_mating$gono_cycle_1`
##             diff         lwr         upr     p adj
## 2-1 -0.027715571 -0.08595200 0.030520860 0.6024690
## 3-1 -0.055912073 -0.11414850 0.002324359 0.0647569
## 4-1 -0.051893418 -0.11012985 0.006343014 0.0987750
## 3-2 -0.028196501 -0.08643293 0.030039931 0.5888318
## 4-2 -0.024177846 -0.08241428 0.034058585 0.7010695
## 4-3  0.004018655 -0.05421778 0.062255086 0.9979222
```

Residuals plot of the transformed Hatch Rate

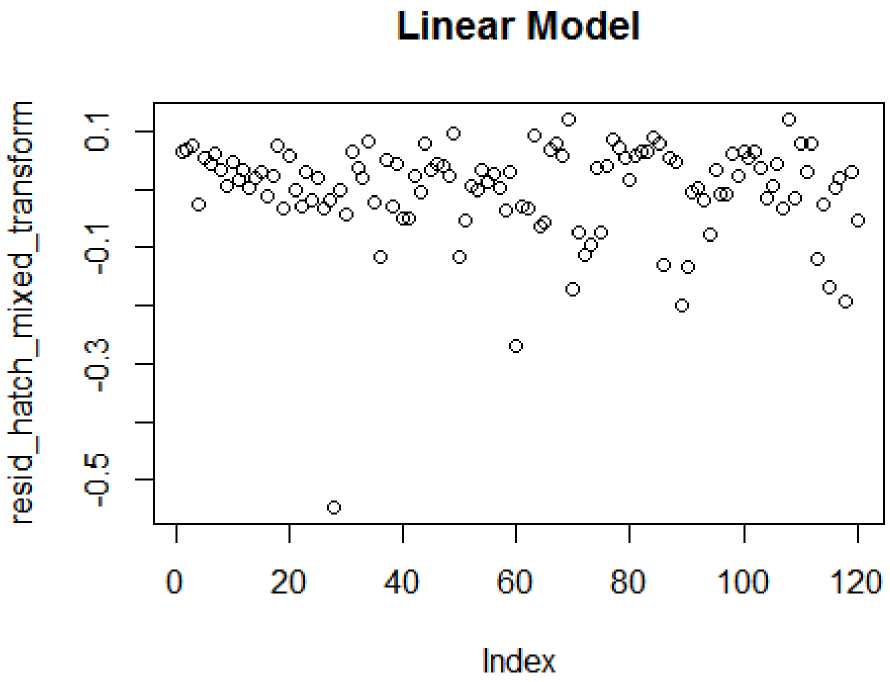

Graph Transformed Hatch Hate

gono_cycle_1	N	avg_value	sd	se
1	30	0.9116497	0.1091163	0.0199218
2	30	0.8839342	0.0719104	0.0131290
3	30	0.8557377	0.0895280	0.0163455
4	30	0.8597563	0.0696810	0.0127220

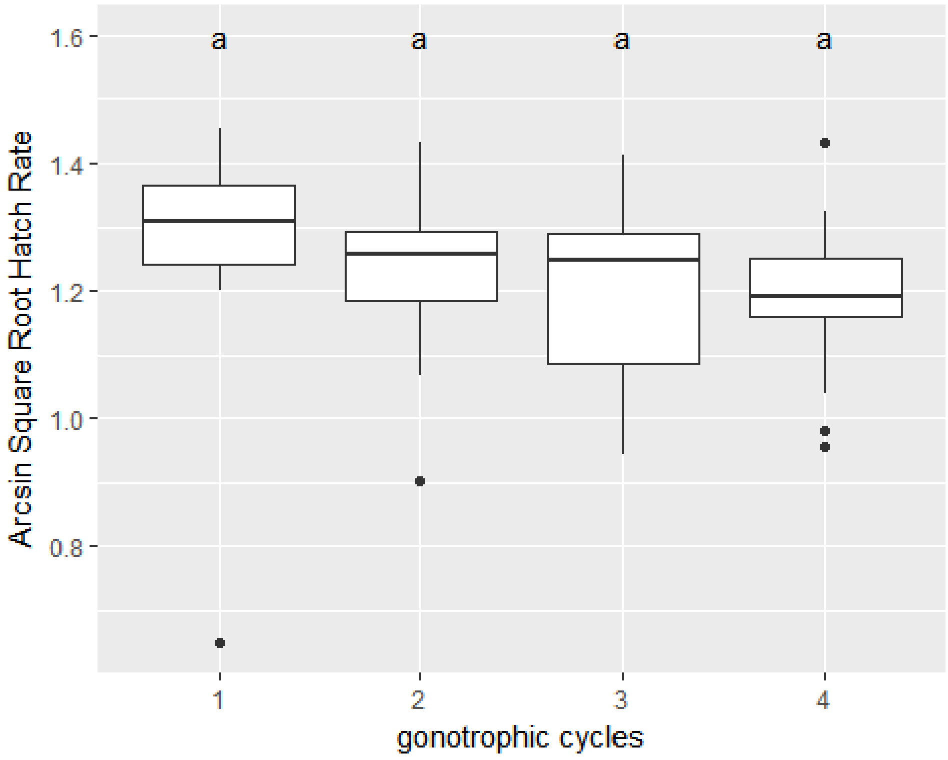

Mean and Standard Deviation

Number of eggs - Summary Table

G. Cycle	N	Mean	SD	SE	Tukey
1	30	137.13	21.91	4.00	b
2	30	128.37	13.81	2.52	ab
3	30	114.03	34.69	6.33	a
4	30	124.53	18.05	3.30	ab

Hatch Rate - Summary Table

G. Cycle	N	Mean	SD	SE	Tukey
1	30	1.29	0.14	0.03	a
2	30	1.24	0.10	0.02	a
3	30	1.20	0.13	0.02	a
4	30	1.20	0.10	0.02	a

The number of eggs per female had a average of 126.0166667 (2.2460667Standard Deviation - SD), while the hatch rate had a 0.8777695(0.0883257SD).

Consecutive Male Exchange

Experimental description in material and methods section.

Normality Test

```
##           n_eggs                        hatch_rate
## statistic 0.9202272                     0.9074054
## p.value   6.620953e-13                  4.859253e-14
## method    "Shapiro-Wilk normality test" "Shapiro-Wilk normality test"
## data.name "X[[i]]"                      "X[[i]]"
```

The Shapiro-Wilk normality test showed that for the number of eggs per female from the consecutive male exchange dataset is nonparametric, as well as the hatch rate with p value 6.620953310^{-13} and 4.859253510^{-14}, respectively. The histogram of each group is presented below.

Histogram and QQ-plot

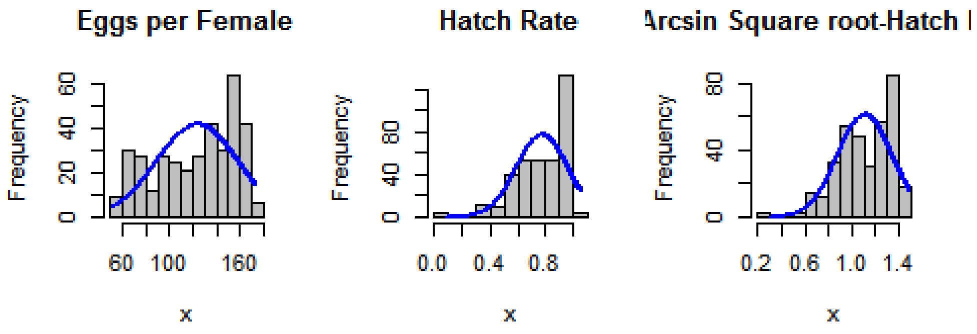

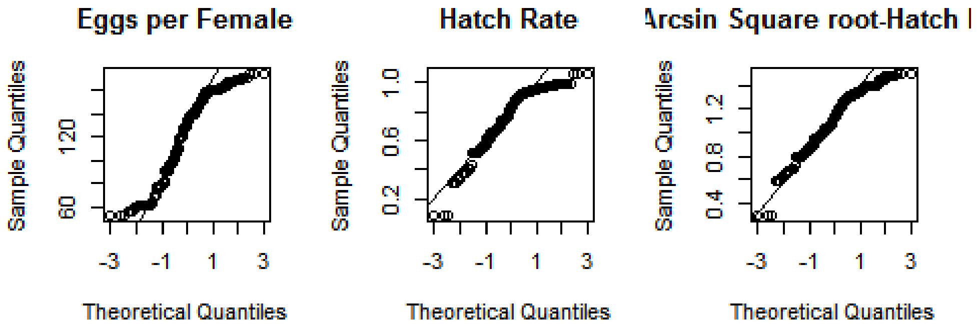

The number of eggs per female can be considered as parametric based ond histogram distribution of frequencies and the plot of sample and theoretical quantiles. However the hatch rate was considered nonparametric using the same approach. For this case an arcsin square root transformation was performed to obtain a parametric distribution.

Linear Model for the number of eggs per female

According to the plot of histogram and QQ-plot, the number of eggs per female was submited to a linear model and check among the different gonotrophic cycles if there was any significant statistical difference among them

```
## [1] "Linear Model"
```

```
##
## Call:
## lm(formula = male_exchange$n_eggs ~ male_exchange$m_cycle)
##
## Residuals:
##    Min     1Q Median     3Q    Max
## -72.90 -24.98   4.25  18.25  66.30
##
## Coefficients:
##                             Estimate Std. Error t value Pr(>|t|)
## (Intercept)                  129.400      5.624  23.007  < 2e-16 ***
## male_exchange$m_cycleWM.1    -5.600      7.954  -0.704 0.481875
## male_exchange$m_cycleTM.2     7.500      7.954   0.943 0.346377
## male_exchange$m_cycleWM.2    -1.200      7.954  -0.151 0.880168
## male_exchange$m_cycleTM.3   -13.000      7.954  -1.634 0.103081
## male_exchange$m_cycleWM.3   -27.500      7.954  -3.457 0.000613 ***
## male_exchange$m_cycleTM.4    17.100      7.954   2.150 0.032256 *
## male_exchange$m_cycleWM.4   -37.100      7.954  -4.664 4.42e-06 ***
## male_exchange$m_cycleTM.5     1.200      7.954   0.151 0.880168
## male_exchange$m_cycleWM.5   -28.700      7.954  -3.608 0.000353 ***
## male_exchange$m_cycleTM.6    15.900      7.954   1.999 0.046387 *
## male_exchange$m_cycleWM.6     4.500      7.954   0.566 0.571928
## ---
## Signif. codes:  0 '***' 0.001 '**' 0.01 '*' 0.05 '.' 0.1 ' ' 1
##
## Residual standard error: 30.81 on 348 degrees of freedom
## Multiple R-squared:  0.2364, Adjusted R-squared:  0.2123
## F-statistic: 9.795 on 11 and 348 DF,  p-value: 1.697e-15
```

```
## [1] "ANOVA"
```

```
##                        Df Sum Sq Mean Sq F value  Pr(>F)
## male_exchange$m_cycle  11 102250    9295   9.795 1.7e-15 ***
## Residuals             348 330252     949
## ---
## Signif. codes:  0 '***' 0.001 '**' 0.01 '*' 0.05 '.' 0.1 ' ' 1
```

```
## [1] "TUKEY - post hoc test"
```

```
##   Tukey multiple comparisons of means
##     95% family-wise confidence level
##
## Fit: aov(formula = n_eggs_m_exchange_lm)
##
## $`male_exchange$m_cycle`
##             diff        lwr         upr     p adj
## WM.1-TM.1   -5.6 -31.772827  20.5728275 0.9999156
## TM.2-TM.1    7.5 -18.672827  33.6728275 0.9986115
## WM.2-TM.1   -1.2 -27.372827  24.9728275 1.0000000
## TM.3-TM.1  -13.0 -39.172827  13.1728275 0.8952499
## WM.3-TM.1  -27.5 -53.672827  -1.3271725 0.0297640
## TM.4-TM.1   17.1  -9.072827  43.2728275 0.5871964
```

```
## WM.4-TM.1 -37.1 -63.272827 -10.9271725 0.0002743
## TM.5-TM.1   1.2 -24.972827  27.3728275 1.0000000
## WM.5-TM.1 -28.7 -54.872827  -2.5271725 0.0180742
## TM.6-TM.1  15.9 -10.272827  42.0728275 0.6939243
## WM.6-TM.1   4.5 -21.672827  30.6728275 0.9999908
## TM.2-WM.1  13.1 -13.072827  39.2728275 0.8902141
## WM.2-WM.1   4.4 -21.772827  30.5728275 0.9999927
## TM.3-WM.1  -7.4 -33.572827  18.7728275 0.9987725
## WM.3-WM.1 -21.9 -48.072827   4.2728275 0.2052329
## TM.4-WM.1  22.7  -3.472827  48.8728275 0.1626657
## WM.4-WM.1 -31.5 -57.672827  -5.3271725 0.0050923
## TM.5-WM.1   6.8 -19.372827  32.9728275 0.9994429
## WM.5-WM.1 -23.1 -49.272827   3.0728275 0.1439876
## TM.6-WM.1  21.5  -4.672827  47.6728275 0.2291798
## WM.6-WM.1  10.1 -16.072827  36.2728275 0.9822979
## WM.2-TM.2  -8.7 -34.872827  17.4728275 0.9948106
## TM.3-TM.2 -20.5 -46.672827   5.6728275 0.2967403
## WM.3-TM.2 -35.0 -61.172827  -8.8271725 0.0008681
## TM.4-TM.2   9.6 -16.572827  35.7728275 0.9881924
## WM.4-TM.2 -44.6 -70.772827 -18.4271725 0.0000027
## TM.5-TM.2  -6.3 -32.472827  19.8728275 0.9997319
## WM.5-TM.2 -36.2 -62.372827 -10.0271725 0.0004530
## TM.6-TM.2   8.4 -17.772827  34.5728275 0.9961700
## WM.6-TM.2  -3.0 -29.172827  23.1728275 0.9999999
## TM.3-WM.2 -11.8 -37.972827  14.3728275 0.9442881
## WM.3-WM.2 -26.3 -52.472827  -0.1271725 0.0476492
## TM.4-WM.2  18.3  -7.872827  44.4728275 0.4780740
## WM.4-WM.2 -35.9 -62.072827  -9.7271725 0.0005341
## TM.5-WM.2   2.4 -23.772827  28.5728275 1.0000000
## WM.5-WM.2 -27.5 -53.672827  -1.3271725 0.0297640
## TM.6-WM.2  17.1  -9.072827  43.2728275 0.5871964
## WM.6-WM.2   5.7 -20.472827  31.8728275 0.9998994
## WM.3-TM.3 -14.5 -40.672827  11.6728275 0.8044148
## TM.4-TM.3  30.1   3.927173  56.2728275 0.0097624
## WM.4-TM.3 -24.1 -50.272827   2.0728275 0.1044303
## TM.5-TM.3  14.2 -11.972827  40.3728275 0.8251228
## WM.5-TM.3 -15.7 -41.872827  10.4728275 0.7108847
## TM.6-TM.3  28.9   2.727173  55.0728275 0.0165883
## WM.6-TM.3  17.5  -8.672827  43.6728275 0.5506724
## TM.4-WM.3  44.6  18.427173  70.7728275 0.0000027
## WM.4-WM.3  -9.6 -35.772827  16.5728275 0.9881924
## TM.5-WM.3  28.7   2.527173  54.8728275 0.0180742
## WM.5-WM.3  -1.2 -27.372827  24.9728275 1.0000000
## TM.6-WM.3  43.4  17.227173  69.5728275 0.0000060
## WM.6-WM.3  32.0   5.827173  58.1728275 0.0040035
## WM.4-TM.4 -54.2 -80.372827 -28.0271725 0.0000000
## TM.5-TM.4 -15.9 -42.072827  10.2728275 0.6939243
## WM.5-TM.4 -45.8 -71.972827 -19.6271725 0.0000012
## TM.6-TM.4  -1.2 -27.372827  24.9728275 1.0000000
## WM.6-TM.4 -12.6 -38.772827  13.5728275 0.9139091
## TM.5-WM.4  38.3  12.127173  64.4728275 0.0001380
```

```
## WM.5-WM.4   8.4 -17.772827  34.5728275 0.9961700
## TM.6-WM.4  53.0  26.827173  79.1728275 0.0000000
## WM.6-WM.4  41.6  15.427173  67.7728275 0.0000188
## WM.5-TM.5 -29.9 -56.072827  -3.7271725 0.0106835
## TM.6-TM.5  14.7 -11.472827  40.8728275 0.7899612
## WM.6-TM.5   3.3 -22.872827  29.4728275 0.9999996
## TM.6-WM.5  44.6  18.427173  70.7728275 0.0000027
## WM.6-WM.5  33.2   7.027173  59.3728275 0.0022099
## WM.6-TM.6 -11.4 -37.572827  14.7728275 0.9562143
```

Ploting Residuals of Number of eggs per female

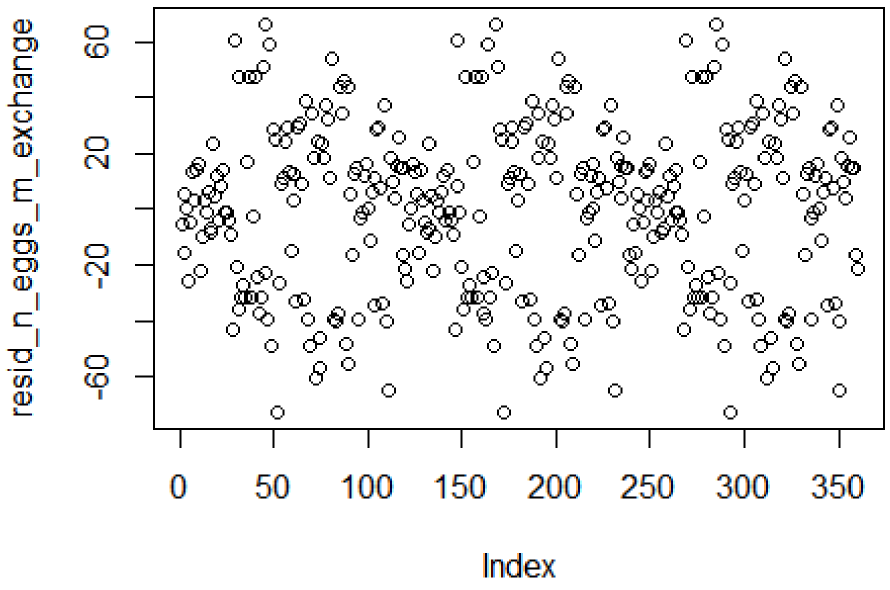

Graph number of eggs per female by gonotrophic Cycles

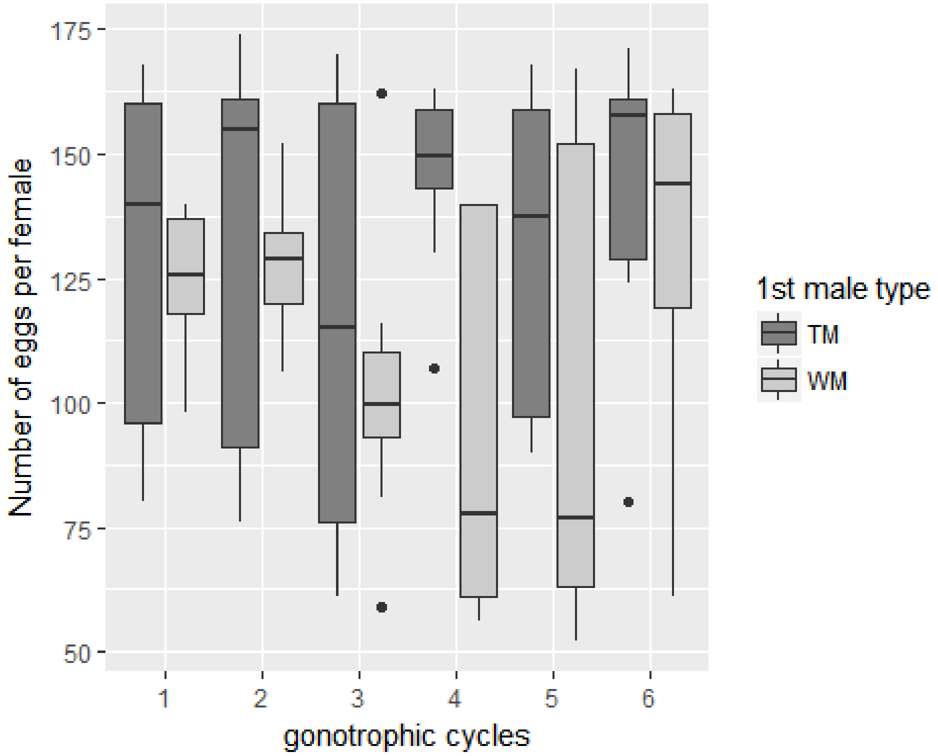

Linear Model for the transformed hatch rate

According to the plot of histogram and QQ-plot, the hatch rate was submited to a arcsin square root transformation and later to a linear model and check among the different gonotrophic cycles if there was any significant statistical difference among them.

```
## [1] "Linear Model"

##
## Call:
## lm(formula = male_exchange$hatch_rate ~ male_exchange$m_cycle)
##
## Residuals:
##      Min       1Q   Median       3Q      Max
## -0.64967 -0.10991  0.04447  0.11928  0.29527
##
## Coefficients:
##                            Estimate Std. Error t value Pr(>|t|)
## (Intercept)                0.790426   0.031576  25.033  < 2e-16 ***
## male_exchange$m_cycleWM.1  0.055331   0.044655   1.239  0.21615
## male_exchange$m_cycleTM.2 -0.098018   0.044655  -2.195  0.02882 *
## male_exchange$m_cycleWM.2 -0.147281   0.044655  -3.298  0.00107 **
## male_exchange$m_cycleTM.3  0.045965   0.044655   1.029  0.30403
## male_exchange$m_cycleWM.3  0.119987   0.044655   2.687  0.00756 **
## male_exchange$m_cycleTM.4 -0.081130   0.044655  -1.817  0.07011 .
## male_exchange$m_cycleWM.4  0.074252   0.044655   1.663  0.09725 .
```

```
## male_exchange$m_cycleTM.5 -0.029360   0.044655  -0.657  0.51130
## male_exchange$m_cycleWM.5 -0.009203   0.044655  -0.206  0.83683
## male_exchange$m_cycleTM.6 -0.025720   0.044655  -0.576  0.56500
## male_exchange$m_cycleWM.6 -0.056841   0.044655  -1.273  0.20391
## ---
## Signif. codes:  0 '***' 0.001 '**' 0.01 '*' 0.05 '.' 0.1 ' ' 1
##
## Residual standard error: 0.1729 on 348 degrees of freedom
## Multiple R-squared:  0.1596, Adjusted R-squared:  0.133
## F-statistic: 6.007 on 11 and 348 DF,  p-value: 5.389e-09
```

```
## [1] "ANOVA"
```

```
##                        Df Sum Sq Mean Sq F value   Pr(>F)
## male_exchange$m_cycle  11  1.976 0.17967   6.007 5.39e-09 ***
## Residuals             348 10.409 0.02991
## ---
## Signif. codes:  0 '***' 0.001 '**' 0.01 '*' 0.05 '.' 0.1 ' ' 1
```

```
##   Tukey multiple comparisons of means
##     95% family-wise confidence level
##
## Fit: aov(formula = hatch_m_exchange_lm)
##
## $`male_exchange$m_cycle`
##                   diff          lwr           upr     p adj
## WM.1-TM.1  0.055331136 -0.091606550  0.2022688217 0.9854132
## TM.2-TM.1 -0.098017672 -0.244955358  0.0489200142 0.5544099
## WM.2-TM.1 -0.147281442 -0.294219128 -0.0003437558 0.0488560
## TM.3-TM.1  0.045965282 -0.100972404  0.1929029675 0.9969422
## WM.3-TM.1  0.119986950 -0.026950736  0.2669246358 0.2371992
## TM.4-TM.1 -0.081129604 -0.228067290  0.0658080816 0.8078808
## WM.4-TM.1  0.074252378 -0.072685308  0.2211900637 0.8836582
## TM.5-TM.1 -0.029359983 -0.176297669  0.1175777025 0.9999575
## WM.5-TM.1 -0.009203351 -0.156141037  0.1377343353 1.0000000
## TM.6-TM.1 -0.025720350 -0.172658036  0.1212173357 0.9999890
## WM.6-TM.1 -0.056840954 -0.203778640  0.0900967321 0.9819571
## TM.2-WM.1 -0.153348807 -0.300286493 -0.0064111216 0.0320663
## WM.2-WM.1 -0.202612577 -0.349550263 -0.0556748916 0.0004813
## TM.3-WM.1 -0.009365854 -0.156303540  0.1375718317 1.0000000
## WM.3-WM.1  0.064655814 -0.082281872  0.2115935000 0.9529490
## TM.4-WM.1 -0.136460740 -0.283398426  0.0104769458 0.0974465
## WM.4-WM.1  0.018921242 -0.128016444  0.1658589279 0.9999996
## TM.5-WM.1 -0.084691119 -0.231628805  0.0622465667 0.7607501
## WM.5-WM.1 -0.064534486 -0.211472172  0.0824031995 0.9535670
## TM.6-WM.1 -0.081051486 -0.227989172  0.0658861999 0.8088586
## WM.6-WM.1 -0.112172090 -0.259109776  0.0347655963 0.3359661
## WM.2-TM.2 -0.049263770 -0.196201456  0.0976739159 0.9944150
## TM.3-TM.2  0.143982953 -0.002954733  0.2909206392 0.0608095
## WM.3-TM.2  0.218004622  0.071066936  0.3649423075 0.0001011
## TM.4-TM.2  0.016888067 -0.130049619  0.1638257533 0.9999999
## WM.4-TM.2  0.172270050  0.025332364  0.3192077354 0.0074685
```

```
## TM.5-TM.2  0.068657688 -0.078279998  0.2155953742 0.9291047
## WM.5-TM.2  0.088814321 -0.058123365  0.2357520070 0.7007652
## TM.6-TM.2  0.072297321 -0.074640364  0.2192350074 0.9012046
## WM.6-TM.2  0.041176718 -0.105760968  0.1881144038 0.9988692
## TM.3-WM.2  0.193246723  0.046309037  0.3401844092 0.0011787
## WM.3-WM.2  0.267268392  0.120330706  0.4142060775 0.0000004
## TM.4-WM.2  0.066151837 -0.080785849  0.2130895233 0.9448344
## WM.4-WM.2  0.221533819  0.074596134  0.3684715054 0.0000697
## TM.5-WM.2  0.117921458 -0.029016228  0.2648591442 0.2613109
## WM.5-WM.2  0.138078091 -0.008859595  0.2850157770 0.0883426
## TM.6-WM.2  0.121561091 -0.025376594  0.2684987774 0.2198276
## WM.6-WM.2  0.090440488 -0.056497198  0.2373781738 0.6758421
## WM.3-TM.3  0.074021668 -0.072916018  0.2209593542 0.8858227
## TM.4-TM.3 -0.127094886 -0.274032572  0.0198428000 0.1656946
## WM.4-TM.3  0.028287096 -0.118650590  0.1752247821 0.9999708
## TM.5-TM.3 -0.075325265 -0.222262951  0.0716124209 0.8732630
## WM.5-TM.3 -0.055168632 -0.202106318  0.0917690537 0.9857516
## TM.6-TM.3 -0.071685632 -0.218623318  0.0752520541 0.9063243
## WM.6-TM.3 -0.102806235 -0.249743921  0.0441314505 0.4769944
## TM.4-WM.3 -0.201116554 -0.348054240 -0.0541788683 0.0005569
## WM.4-WM.3 -0.045734572 -0.192672258  0.1012031138 0.9970753
## TM.5-WM.3 -0.149346933 -0.296284619 -0.0024092474 0.0424440
## WM.5-WM.3 -0.129190300 -0.276127986  0.0177473854 0.1479684
## TM.6-WM.3 -0.145707300 -0.292644986  0.0012303858 0.0542839
## WM.6-WM.3 -0.176827904 -0.323765590 -0.0298902178 0.0050997
## WM.4-TM.4  0.155381982  0.008444296  0.3023196680 0.0277017
## TM.5-TM.4  0.051769621 -0.095168065  0.1987073068 0.9915154
## WM.5-TM.4  0.071926254 -0.075011432  0.2188639396 0.9043313
## TM.6-TM.4  0.055409254 -0.091528432  0.2023469400 0.9852483
## WM.6-TM.4  0.024288650 -0.122649035  0.1712263364 0.9999939
## TM.5-WM.4 -0.103612361 -0.250550047  0.0433253247 0.4641736
## WM.5-WM.4 -0.083455728 -0.230393414  0.0634819575 0.7776434
## TM.6-WM.4 -0.099972728 -0.246910414  0.0469649579 0.5226275
## WM.6-WM.4 -0.131093332 -0.278031018  0.0158443543 0.1331395
## WM.5-TM.5  0.020156633 -0.126781053  0.1670943187 0.9999991
## TM.6-TM.5  0.003639633 -0.143298053  0.1505773191 1.0000000
## WM.6-TM.5 -0.027480970 -0.174418656  0.1194567155 0.9999782
## TM.6-WM.5 -0.016517000 -0.163454686  0.1304206863 0.9999999
## WM.6-WM.5 -0.047637603 -0.194575289  0.0993000827 0.9958177
## WM.6-TM.6 -0.031120604 -0.178058290  0.1158170823 0.9999238
```

Ploting Residuals of Transformed Hatch Rate

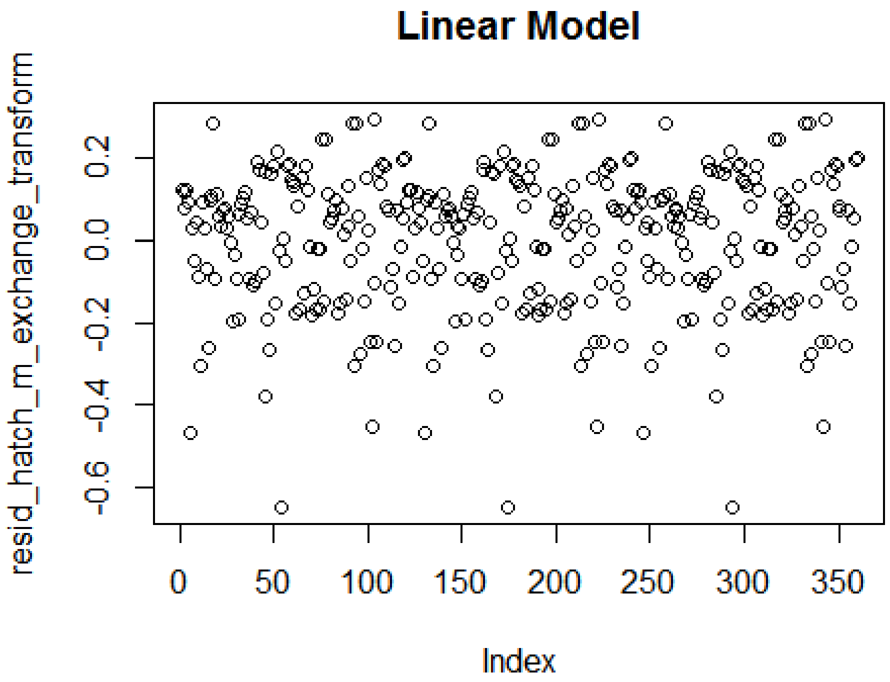

Graph Transformed Hatch Hate

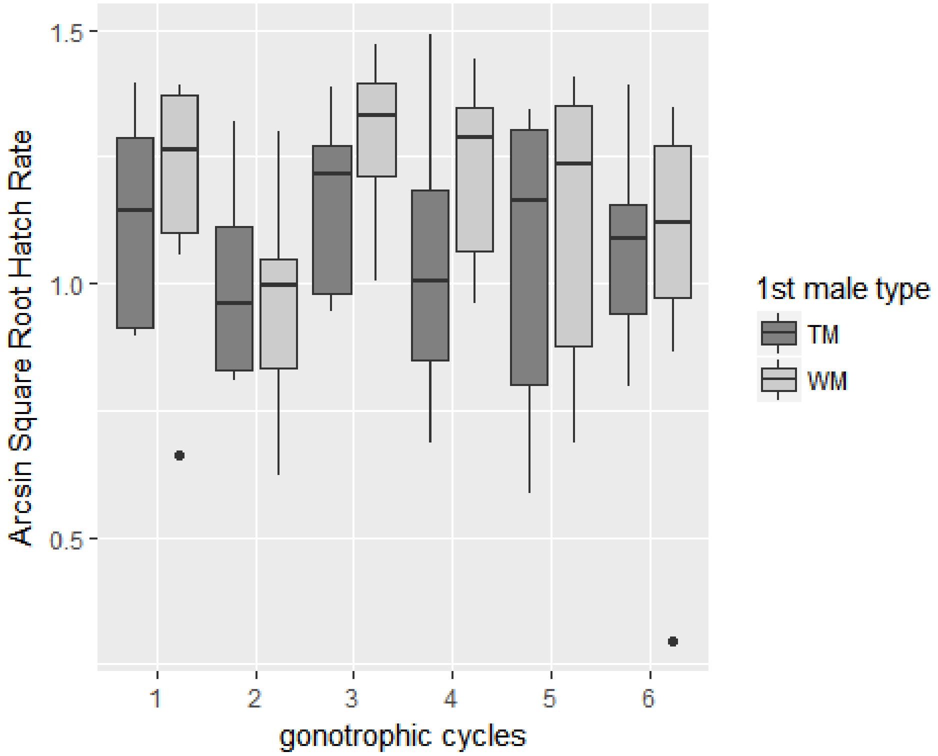

Mean and Standard Deviation

Number of eggs - Summary Table

G. Cycle	N	Mean	SD	SE	Tukey
TM.1	30	129.4	33.56	6.13	ab
WM.1	30	123.8	13.06	2.38	ab
TM.2	30	136.9	37.17	6.79	acd
WM.2	30	128.2	12.41	2.26	b
TM.3	30	116.4	45.42	8.29	ab
WM.3	30	101.9	25.46	4.65	b
TM.4	30	146.5	16.55	3.02	abc
WM.4	30	92.3	35.40	6.46	ab
TM.5	30	130.6	27.68	5.05	cd
WM.5	30	100.7	44.15	8.06	d
TM.6	30	145.3	26.48	4.84	cd
WM.6	30	133.9	30.01	5.48	ab

Hatch Rate - Summary Table

G. Cycle	N	Mean	SD	SE	Tukey
TM.1	30	0.79	0.14	0.03	abce
WM.1	30	0.85	0.17	0.03	de
TM.2	30	0.69	0.15	0.03	abce
WM.2	30	0.64	0.18	0.03	bde
TM.3	30	0.84	0.11	0.02	bcde
WM.3	30	0.91	0.09	0.02	abcde
TM.4	30	0.71	0.20	0.04	abc
WM.4	30	0.86	0.11	0.02	d
TM.5	30	0.76	0.24	0.04	a
WM.5	30	0.78	0.21	0.04	ac
TM.6	30	0.76	0.14	0.03	abcde
WM.6	30	0.73	0.25	0.05	bcde

The number of eggs per female had a average of 123.825 (1.8293448SD), while the hatch rate had a 0.7777579(0.1857411SD).

Pre-determined copulation interval of 20 minutes

Experimental description in material and methods section.

Normality Test

```
##           n_eggs                         hatch_rate
## statistic 0.9828606                      0.9052291
## p.value   0.5606423                      0.0002051057
## method    "Shapiro-Wilk normality test" "Shapiro-Wilk normality test"
## data.name "X[[i]]"                       "X[[i]]"
```

The Shapiro-Wilk normality test showed that for the number of eggs per female from the consecutive male exchange dataset is nonparametric, as well as the hatch rate with p value 0.5606423 and 2.051057310^{-4}, respectively. The histogram of each group is presented below.

Histogram and QQ-plot

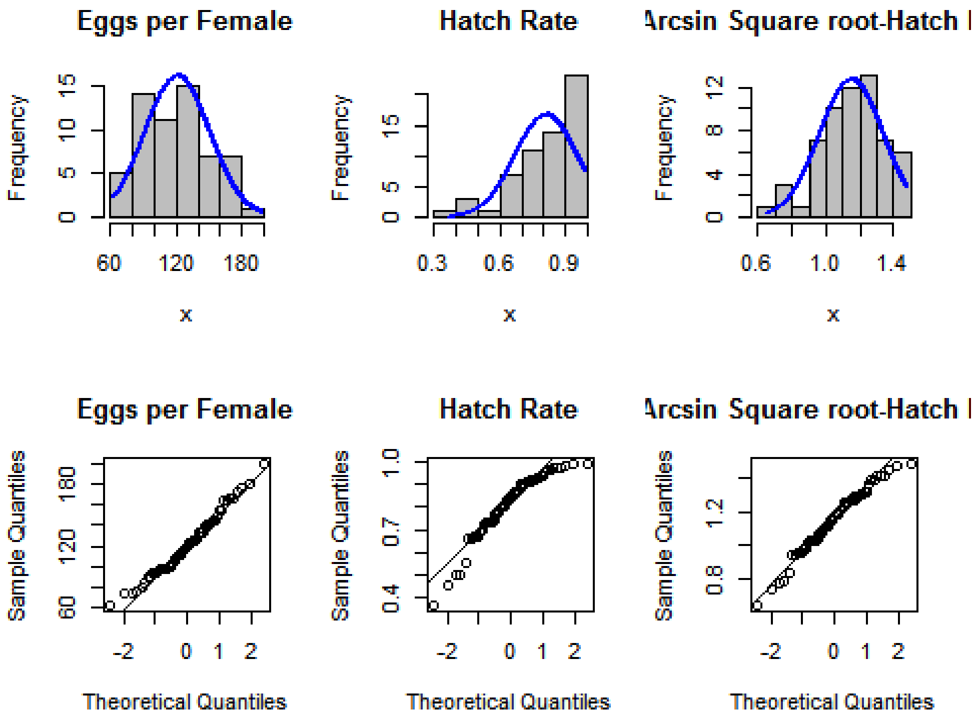

The number of eggs per female can be considered as parametric based ond histogram distribution of frequencies and the plot of sample and theoretical quantiles. However the hatch rate was considered nonparametric using the same approach. For this case an arcsin square root transformation was performed to obtain a parametric distribution.

Linear Model for the number of eggs per female

According to the plot of histogram and QQ-plot, the number of eggs per female was submited to a linear model and check among the different gonotrophic cycles if there was any significant statistical difference among them

```
## [1] "Linear Model"
```

```
##
## Call:
## lm(formula = interval_20$n_eggs ~ interval_20$poly_cycle)
##
## Residuals:
##     Min      1Q  Median      3Q     Max
## -59.722 -21.736   0.299  17.507  77.278
##
## Coefficients:
##                           Estimate Std. Error t value Pr(>|t|)
## (Intercept)                108.250     10.607  10.205 3.31e-14 ***
## interval_20$poly_cycle2.a   23.375     15.001   1.558    0.125
## interval_20$poly_cycle1.b   27.500     18.372   1.497    0.140
## interval_20$poly_cycle2.b   23.500     18.372   1.279    0.206
## interval_20$poly_cycle1.c    8.528     12.748   0.669    0.506
## interval_20$poly_cycle2.c   13.472     12.748   1.057    0.295
## ---
## Signif. codes:  0 '***' 0.001 '**' 0.01 '*' 0.05 '.' 0.1 ' ' 1
##
## Residual standard error: 30 on 54 degrees of freedom
## Multiple R-squared:  0.07354,    Adjusted R-squared:  -0.01224
## F-statistic: 0.8573 on 5 and 54 DF,  p-value: 0.5157

## [1] "ANOVA"

##                        Df Sum Sq Mean Sq F value Pr(>F)
## interval_20$poly_cycle  5   3858   771.7   0.857  0.516
## Residuals              54  48606   900.1

## [1] "TUKEY - post hoc test"

##   Tukey multiple comparisons of means
##     95% family-wise confidence level
##
## Fit: aov(formula = n_eggs_interval_lm)
##
## $`interval_20$poly_cycle`
##                diff       lwr      upr     p adj
## 2.a-1.a  23.375000 -20.94475 67.69475 0.6288695
## 1.b-1.a  27.500000 -26.78038 81.78038 0.6677068
## 2.b-1.a  23.500000 -30.78038 77.78038 0.7949464
## 1.c-1.a   8.527778 -29.13676 46.19232 0.9846124
## 2.c-1.a  13.472222 -24.19232 51.13676 0.8960505
## 1.b-2.a   4.125000 -50.15538 58.40538 0.9999171
## 2.b-2.a   0.125000 -54.15538 54.40538 1.0000000
## 1.c-2.a -14.847222 -52.51176 22.81732 0.8514278
## 2.c-2.a  -9.902778 -47.56732 27.76176 0.9703004
## 2.b-1.b  -4.000000 -66.67759 58.67759 0.9999651
## 1.c-1.b -18.972222 -67.96955 30.02510 0.8606455
## 2.c-1.b -14.027778 -63.02510 34.96955 0.9573390
## 1.c-2.b -14.972222 -63.96955 34.02510 0.9441304
```

```
## 2.c-2.b -10.027778 -59.02510 38.96955 0.9902603
## 2.c-1.c   4.944444 -24.60205 34.49094 0.9961767
```

Residuals plot of the number of eggs per female

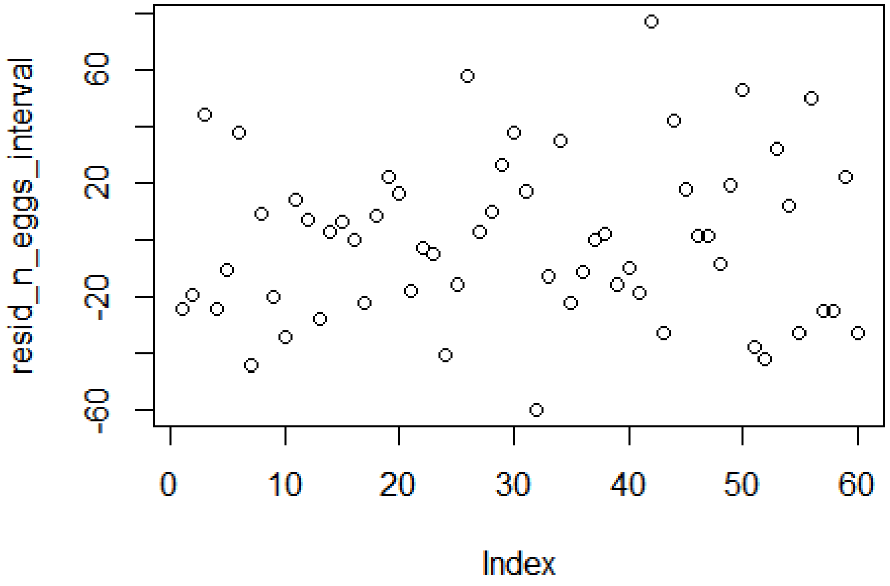

Graph number of eggs per female by gonotrophic Cycles

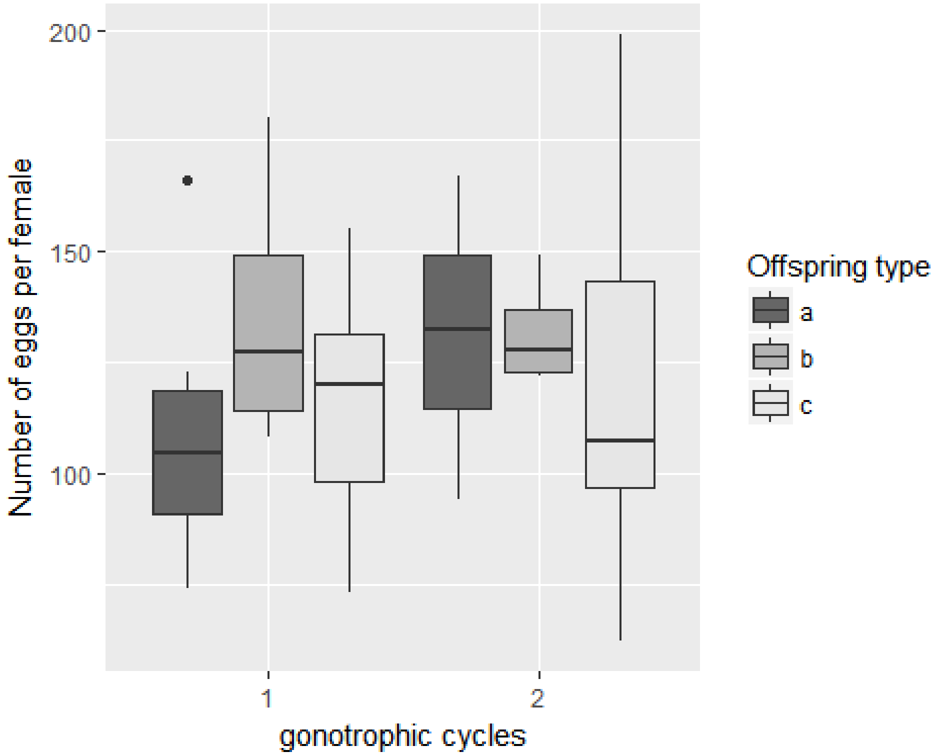

Linear Model for the transformed hatch rate

According to the plot of histogram and QQ-plot, the hatch rate was submited to a arcsin square root transformation and later to a linear model and check among the different gonotrophic cycles if there was any significant statistical difference among them.

```
## [1] "Linear Model"

##
## Call:
## lm(formula = interval_20$hatch_rate ~ interval_20$poly_cycle)
##
## Residuals:
##      Min       1Q   Median       3Q      Max
## -0.43429 -0.07646  0.02713  0.10660  0.19272
##
## Coefficients:
##                           Estimate Std. Error t value Pr(>|t|)
## (Intercept)                0.79573    0.05124  15.529   <2e-16 ***
## interval_20$poly_cycle2.a  0.02097    0.07246   0.289    0.773
## interval_20$poly_cycle1.b -0.03162    0.08875  -0.356    0.723
## interval_20$poly_cycle2.b  0.14352    0.08875   1.617    0.112
## interval_20$poly_cycle1.c  0.01165    0.06158   0.189    0.851
## interval_20$poly_cycle2.c  0.01473    0.06158   0.239    0.812
## ---
## Signif. codes:  0 '***' 0.001 '**' 0.01 '*' 0.05 '.' 0.1 ' ' 1
```

```
##
## Residual standard error: 0.1449 on 54 degrees of freedom
## Multiple R-squared:  0.06314,    Adjusted R-squared:  -0.02361
## F-statistic: 0.7279 on 5 and 54 DF,  p-value: 0.6056

## [1] "ANOVA"

##                         Df Sum Sq Mean Sq F value Pr(>F)
## interval_20$poly_cycle  5 0.0764 0.01529   0.728  0.606
## Residuals              54 1.1342 0.02100

##   Tukey multiple comparisons of means
##     95% family-wise confidence level
##
## Fit: aov(formula = hatch_interval_lm)
##
## $`interval_20$poly_cycle`
##                 diff        lwr       upr     p adj
## 2.a-1.a  0.020966593 -0.1931282 0.2350614 0.9997111
## 1.b-1.a -0.031619934 -0.2938315 0.2305916 0.9992032
## 2.b-1.a  0.143515565 -0.1186960 0.4057271 0.5911498
## 1.c-1.a  0.011651059 -0.1702945 0.1935967 0.9999645
## 2.c-1.a  0.014731791 -0.1672138 0.1966774 0.9998866
## 1.b-2.a -0.052586527 -0.3147981 0.2096250 0.9911244
## 2.b-2.a  0.122548972 -0.1396626 0.3847605 0.7381101
## 1.c-2.a -0.009315534 -0.1912611 0.1726301 0.9999883
## 2.c-2.a -0.006234802 -0.1881804 0.1757108 0.9999984
## 2.b-1.b  0.175135499 -0.1276403 0.4779113 0.5320430
## 1.c-1.b  0.043270993 -0.1934197 0.2799617 0.9942148
## 2.c-1.b  0.046351725 -0.1903390 0.2830424 0.9920445
## 1.c-2.b -0.131864506 -0.3685552 0.1048262 0.5725254
## 2.c-2.b -0.128783774 -0.3654745 0.1079069 0.5972809
## 2.c-1.c  0.003080731 -0.1396491 0.1458106 0.9999998
```

Residuals plot of the Transformed Hatch Rate

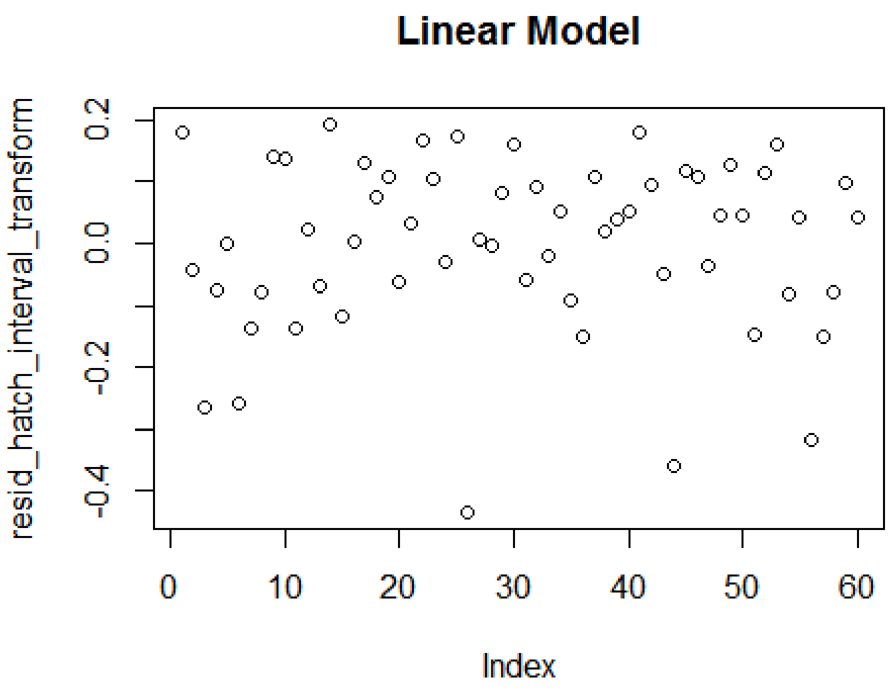

Graph Transformed Hatch Hate

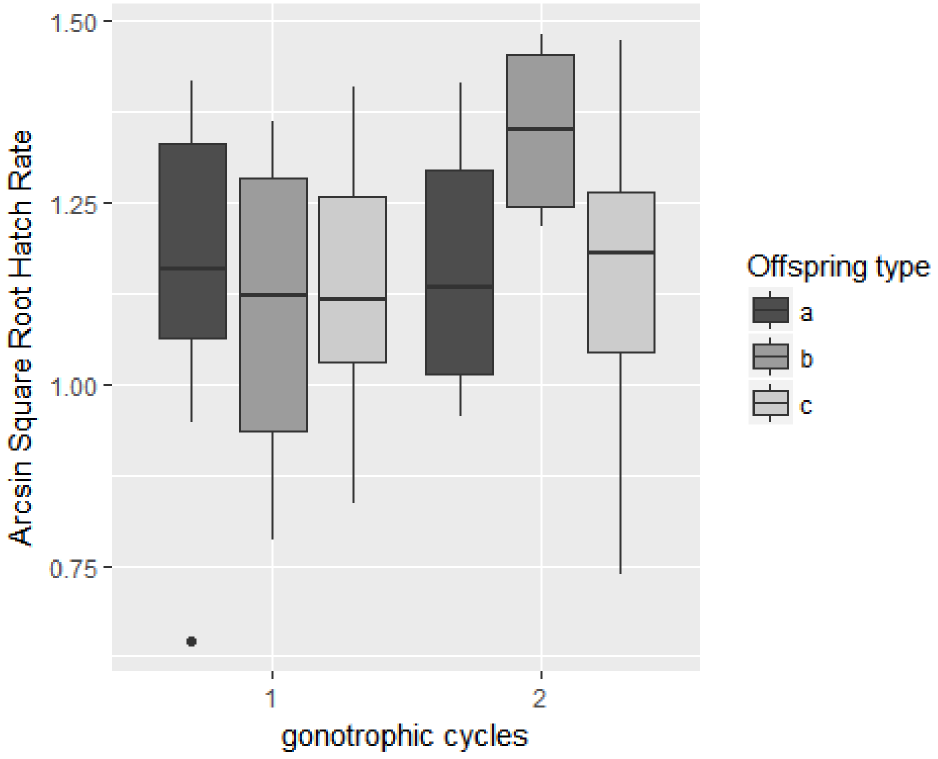

Mean and Standard Deviation

Number of eggs - Summary Table

G. Cycle	N	Mean	SD	SE	Tukey
1.a	8	108.25	28.62	10.12	a
2.a	8	131.62	26.91	9.51	a
1.b	4	135.75	32.29	16.15	a
2.b	4	131.75	12.53	6.26	a
1.c	18	116.78	24.21	5.71	a
2.c	18	121.72	37.76	8.90	a

Hatch rate - Summary Table

G. Cycle	N	Mean	SD	SE	Tukey
1.a	8	1.14	0.26	0.09	a
2.a	8	1.15	0.17	0.06	a
1.b	4	1.10	0.26	0.13	a
2.b	4	1.35	0.13	0.07	a
1.c	18	1.14	0.15	0.04	a
2.c	18	1.15	0.19	0.04	a

The number of eggs per female had a average of 121.3666667 (3.8497169SD), while the hatch rate had a 0.8139029(0.1432481SD).

Interrupted coitus

Experimental description in material and methods section.

Normality Test

```
##           n_eggs                        hatch_rate
## statistic 0.9572362                     0.9561252
## p.value   2.720539e-06                  2.048405e-06
## method    "Shapiro-Wilk normality test" "Shapiro-Wilk normality test"
## data.name "X[[i]]"                      "X[[i]]"
```

The Shapiro-Wilk normality test showed that for the number of eggs per female from the consecutive male exchange dataset is nonparametric, as well as the hatch rate with p value 2.720538810^{-6} and 2.048405210^{-6}, respectively. The histogram of each group is presented below.

Histogram and QQ-plot

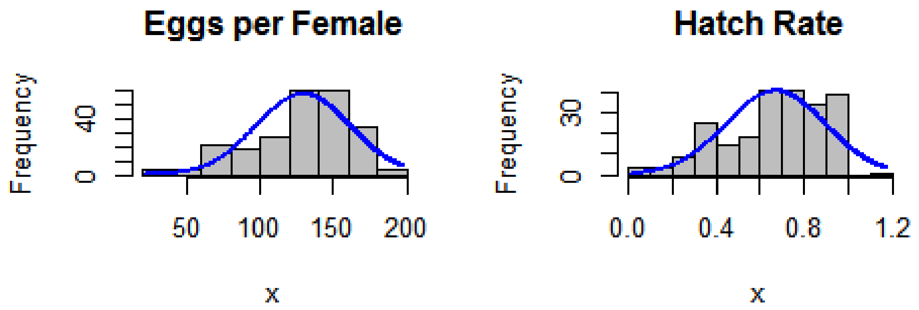

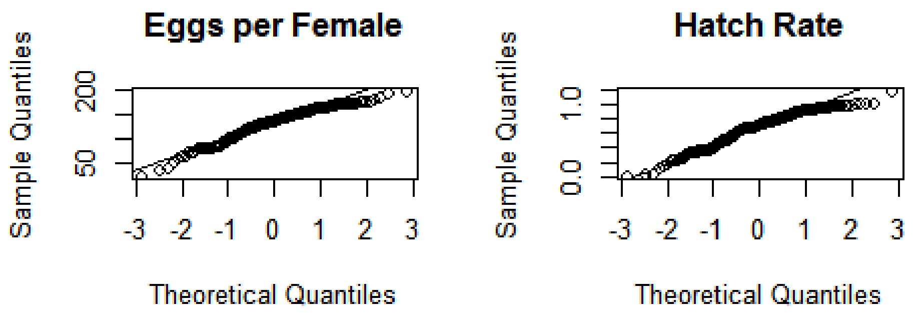

The number of eggs per female can be considered as parametric based ond histogram distribution of frequencies and the plot of sample and theoretical quantiles. Simirlaly, for the hatch rate which performed a parametric distribution.

Linear Model for the number of eggs per female

According to the plot of histogram and QQ-plot, the number of eggs per female was submited to a linear model and check among the different gonotrophic cycles if there was any significant statistical difference among them

```
## [1] "Linear Model"

## Call:
## lm(formula = interrupted_coitus$n_eggs ~ interrupted_coitus$mating_male)
##
## Residuals:
##     Min      1Q  Median      3Q     Max
## -102.09  -19.63    4.20   25.08   73.91
##
## Coefficients:
##                                    Estimate Std. Error t value Pr(>|t|)
## (Intercept)                         130.366      4.996  26.093   <2e-16
## interrupted_coitus$mating_maleWM.A    4.397      7.204   0.610    0.542
## interrupted_coitus$mating_maleTM.B   -2.566      7.362  -0.349    0.728
## interrupted_coitus$mating_maleWM.B   -7.280      7.362  -0.989    0.324
## interrupted_coitus$mating_maleTM.C    3.089      6.944   0.445    0.657
```

```
## interrupted_coitus$mating_maleWM.C   -4.807      7.421  -0.648    0.518
##
## (Intercept)                        ***
## interrupted_coitus$mating_maleWM.A
## interrupted_coitus$mating_maleTM.B
## interrupted_coitus$mating_maleWM.B
## interrupted_coitus$mating_maleTM.C
## interrupted_coitus$mating_maleWM.C
## ---
## Signif. codes:  0 '***' 0.001 '**' 0.01 '*' 0.05 '.' 0.1 ' ' 1
##
## Residual standard error: 31.99 on 221 degrees of freedom
## Multiple R-squared:  0.0167, Adjusted R-squared:  -0.005551
## F-statistic: 0.7505 on 5 and 221 DF,  p-value: 0.5865
```

```
## [1] "ANOVA"
```

```
##                                 Df Sum Sq Mean Sq F value Pr(>F)
## interrupted_coitus$mating_male   5   3840   768.1    0.75  0.587
## Residuals                      221 226184  1023.5
```

```
## [1] "TUKEY - post hoc test"
```

```
##   Tukey multiple comparisons of means
##     95% family-wise confidence level
##
## Fit: aov(formula = n_eggs_coitus_lm)
##
## $`interrupted_coitus$mating_male`
##                  diff       lwr       upr     p adj
## WM.A-TM.A   4.397304 -16.31300 25.107609 0.9902049
## TM.B-TM.A  -2.565854 -23.73179 18.600084 0.9993164
## WM.B-TM.A  -7.280139 -28.44608 13.885798 0.9211433
## TM.C-TM.A   3.088692 -16.87532 23.052706 0.9977827
## WM.C-TM.A  -4.807030 -26.14023 16.526165 0.9871514
## TM.B-WM.A  -6.963158 -28.51043 14.584113 0.9386626
## WM.B-WM.A -11.677444 -33.22471  9.869828 0.6270246
## TM.C-WM.A  -1.308612 -21.67648 19.059250 0.9999700
## WM.C-WM.A  -9.204334 -30.91593 12.507257 0.8273490
## WM.B-TM.B  -4.714286 -26.69985 17.271280 0.9897523
## TM.C-TM.B   5.654545 -15.17644 26.485533 0.9706033
## WM.C-TM.B  -2.241176 -24.38781 19.905458 0.9997171
## TM.C-WM.B  10.368831 -10.46216 31.199818 0.7080988
## WM.C-WM.B   2.473109 -19.67353 24.619744 0.9995419
## WM.C-TM.C  -7.895722 -28.89663 13.105191 0.8885980
```

Residuals plot of the number of eggs per female

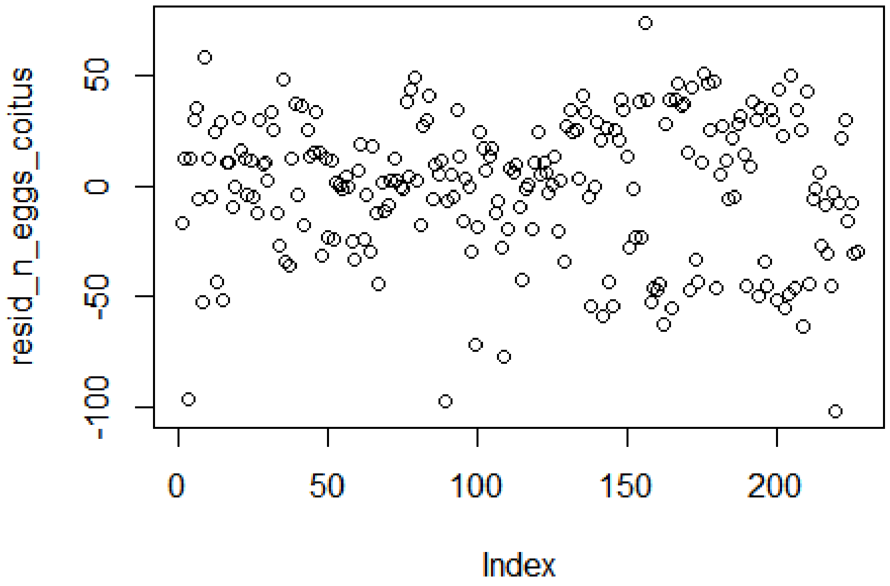

Graph number of eggs per female by gonotrophic Cycles

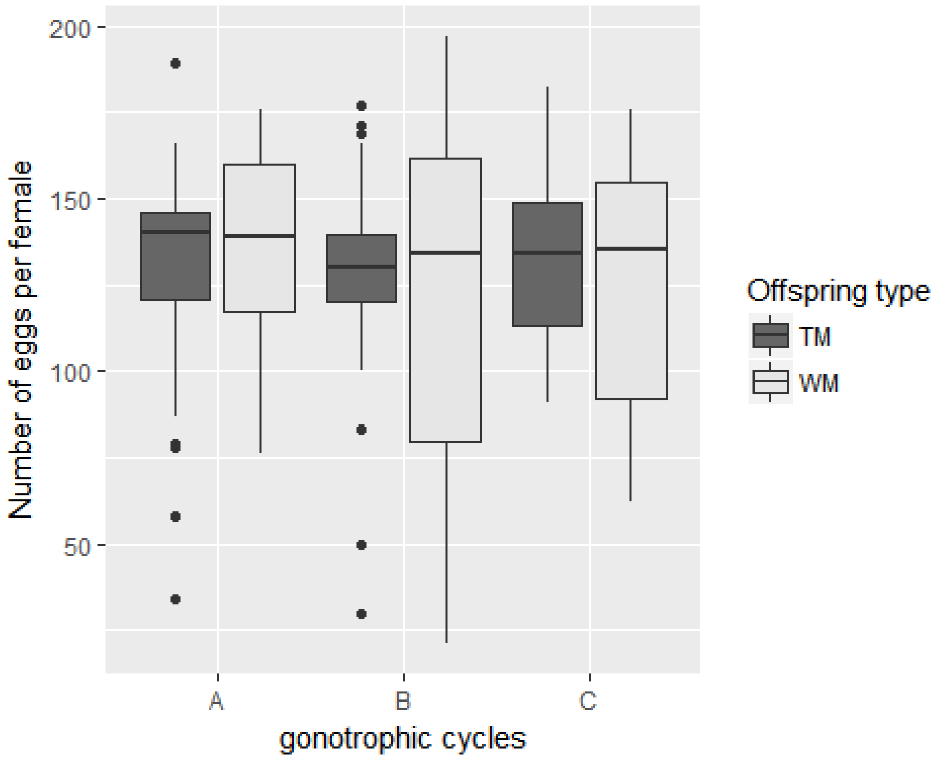

Linear Model for the transformed hatch rate

According to the plot of histogram and QQ-plot, the hatch rate was submited to a arcsin square root transformation and later to a linear model and check among the different gonotrophic cycles if there was any significant statistical difference among them.

```
## [1] "Linear Model"

##
## Call:
## lm(formula = interrupted_coitus$hatch_rate ~
interrupted_coitus$mating_male)
##
## Residuals:
##     Min      1Q  Median      3Q     Max
## -0.7358 -0.1504  0.0276  0.1657  0.5075
##
## Coefficients:
##                                     Estimate Std. Error t value Pr(>|t|)
## (Intercept)                         0.744682   0.034097  21.840  < 2e-16
## interrupted_coitus$mating_maleWM.A -0.118498   0.049163  -2.410 0.016759
## interrupted_coitus$mating_maleTM.B -0.184450   0.050245  -3.671 0.000303
## interrupted_coitus$mating_maleWM.B -0.003984   0.050245  -0.079 0.936872
## interrupted_coitus$mating_maleTM.C -0.075706   0.047392  -1.597 0.111596
## interrupted_coitus$mating_maleWM.C -0.072960   0.050642  -1.441 0.151087
##
## (Intercept)                        ***
## interrupted_coitus$mating_maleWM.A *
## interrupted_coitus$mating_maleTM.B ***
## interrupted_coitus$mating_maleWM.B
## interrupted_coitus$mating_maleTM.C
## interrupted_coitus$mating_maleWM.C
## ---
## Signif. codes:  0 '***' 0.001 '**' 0.01 '*' 0.05 '.' 0.1 ' ' 1
##
## Residual standard error: 0.2183 on 221 degrees of freedom
## Multiple R-squared:  0.07858,    Adjusted R-squared:  0.05773
## F-statistic:  3.77 on 5 and 221 DF,  p-value: 0.002696

## [1] "ANOVA"

##                                 Df Sum Sq Mean Sq F value Pr(>F)
## interrupted_coitus$mating_male   5  0.898 0.17968    3.77 0.0027 **
## Residuals                      221 10.535 0.04767
## ---
## Signif. codes:  0 '***' 0.001 '**' 0.01 '*' 0.05 '.' 0.1 ' ' 1

##   Tukey multiple comparisons of means
##     95% family-wise confidence level
##
## Fit: aov(formula = hatch_coitus_lm)
##
```

```
## $`interrupted_coitus$mating_male`
##                     diff         lwr         upr     p adj
## WM.A-TM.A -0.118497880 -0.25983772  0.02284196 0.1571607
## TM.B-TM.A -0.184450419 -0.32889978 -0.04000106 0.0040594
## WM.B-TM.A -0.003984048 -0.14843341  0.14046531 0.9999996
## TM.C-TM.A -0.075705869 -0.21195256  0.06054083 0.6011213
## WM.C-TM.A -0.072959672 -0.21855050  0.07263116 0.7021034
## TM.B-WM.A -0.065952540 -0.21300436  0.08109928 0.7906340
## WM.B-WM.A  0.114513831 -0.03253798  0.26156565 0.2243853
## TM.C-WM.A  0.042792011 -0.09621080  0.18179482 0.9497864
## WM.C-WM.A  0.045538208 -0.10263503  0.19371145 0.9501380
## WM.B-TM.B  0.180466371  0.03042337  0.33050938 0.0084647
## TM.C-TM.B  0.108744551 -0.03341890  0.25090800 0.2423499
## WM.C-TM.B  0.111490748 -0.03965149  0.26263298 0.2804714
## TM.C-WM.B -0.071721821 -0.21388527  0.07044163 0.6960606
## WM.C-WM.B -0.068975624 -0.22011786  0.08216661 0.7781968
## WM.C-TM.C  0.002746197 -0.14057693  0.14606933 0.9999999
```

Residuals plot of the Transformed Hatch Rate

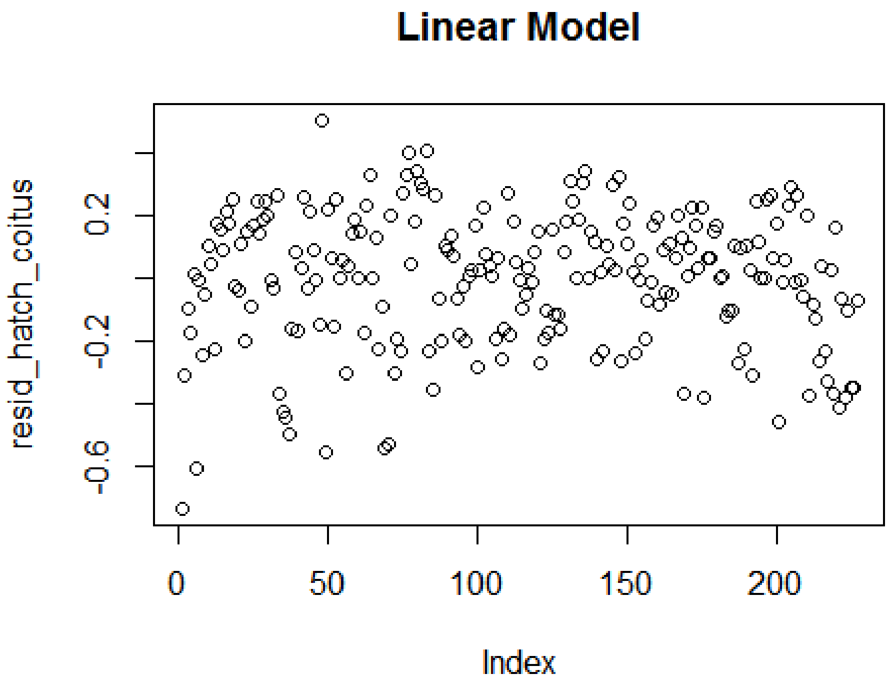

Graph Transformed Hatch Hate

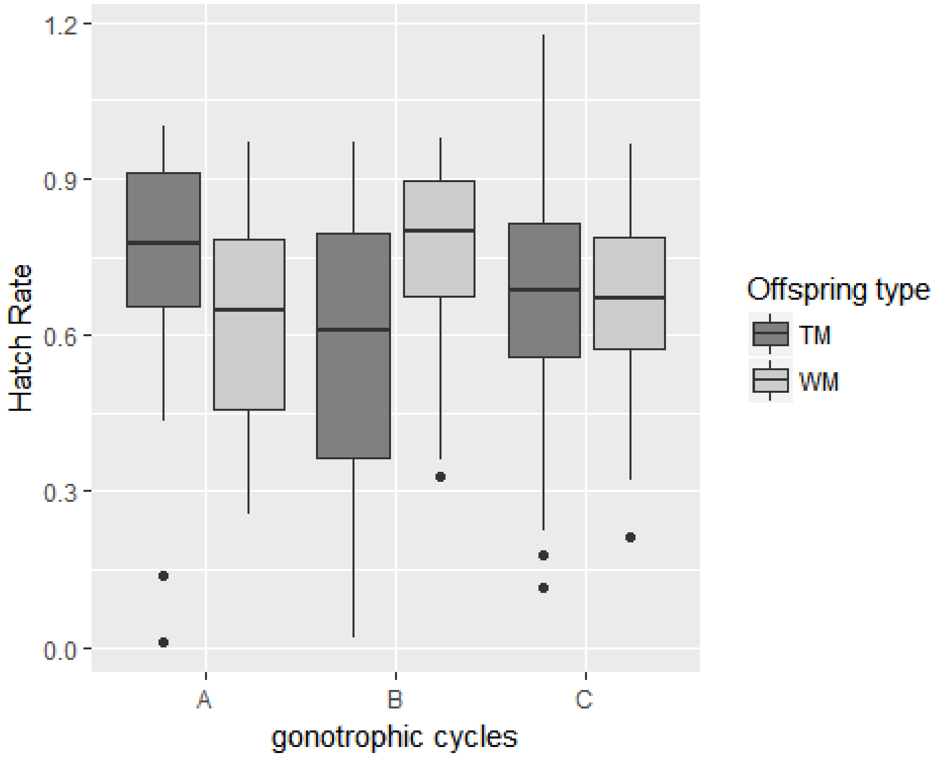

Mean and Standard Deviation

Number of eggs - Summary Table

Mating Group	N	Mean	SD	SE	Tukey
TM.A	41	130.37	29.83	4.66	a
WM.A	38	134.76	29.01	4.71	a
TM.B	35	127.80	30.02	5.07	a
WM.B	35	123.09	43.39	7.33	a
TM.C	44	133.45	23.06	3.48	a
WM.C	34	125.56	35.65	6.11	a

Hatch rate - Summary Table

Mating Group	N	Mean	SD	SE	Tukey
TM.A	41	0.74	0.22	0.03	b
WM.A	38	0.63	0.20	0.03	a
TM.B	35	0.56	0.26	0.04	ab
WM.B	35	0.74	0.19	0.03	ab
TM.C	44	0.67	0.23	0.03	b
WM.C	34	0.67	0.20	0.03	ab

The number of eggs per female had a average of 129.4625551 (2.1174825SD), while the hatch rate had a 0.6701896(0.2249191SD).

Ordered Logistic Regression Model

General descriptive table with total number of crosses in for each mating group according to the first male type

```
##                      mating_time  A  B  C
## male_type1 polyandry
## 1          1                     41 18  1
##            2                      0 14 11
##            3                      0  3 32
## 2          1                     37 15  1
##            2                      1 16  5
##            3                      0  4 28
```

Ordered Logistic Regression

```
##                 Value Std. Error   t value
## polyandry   3.3735882  0.3434941  9.821385
## male_type1 -0.3690141  0.3149059 -1.171823
## A|B         3.5599521  0.6073622  5.861333
## B|C         7.0867917  0.8661780  8.181681
```

```
##                 Value Std. Error   t value      p value
## polyandry   3.3735882  0.3434941  9.821385 9.108340e-23
## male_type1 -0.3690141  0.3149059 -1.171823 2.412680e-01
## A|B         3.5599521  0.6073622  5.861333 4.591673e-09
## B|C         7.0867917  0.8661780  8.181681 2.799124e-16
```

```
## Waiting for profiling to be done...
```

```
##                 2.5 %   97.5 %
## polyandry   2.7530709 4.112016
## male_type1 -0.9931706 0.244955
```

```
##                 2.5 %    97.5 %
## polyandry   2.7003521 4.0468244
## male_type1 -0.9862184 0.2481901
```

```
## Call:
## polr(formula = mating_time ~ polyandry + male_type1, data = coitus,
##     Hess = TRUE)
##
## Coefficients:
##  polyandry male_type1
##  3.3735882 -0.3690141
##
## Intercepts:
##      A|B      B|C
## 3.559952 7.086792
##
```

```
## Residual Deviance: 271.5139
## AIC: 279.5139
```

Odds ratios (OR) and CI

```
##  polyandry male_type1
## 29.1830550  0.6914156
```

```
##                    OR      2.5 %    97.5 %
## polyandry  29.1830550 15.6907433 61.069725
## male_type1  0.6914156  0.3704005  1.277564
```

Probabilities

```
##                            Level          A           B           C
## male_type2 polyandry_fac
## Transgenic Male 1                48.61731451  1.34091664  0.04176885
##            Male 1&2              27.32282951 21.48626591  1.19090458
##            Male 2                 1.98246670 27.22238664 20.79514667
## Wild-type  Male 1                49.03576129  0.93535163  0.02888708
##            Male 1&2              31.76912141 17.40137175  0.82950684
##            Male 2                 2.81740129 30.68751059 16.49508812
```

Plot probabilities

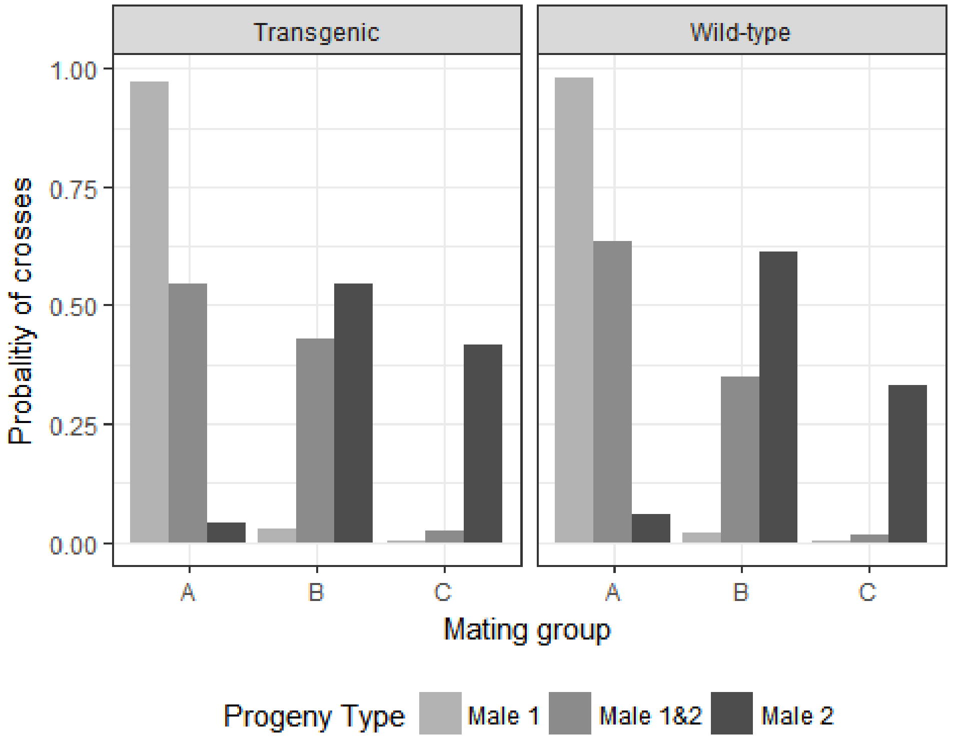

This code was obtained in: https://rpubs.com/rslbliss/r_logistic_ws

Citation: Blissett, R. 2017. Logistic, Ordinal, and Multinomial Regression in R. UCLA: Statistical Consulting Group. https://rpubs.com/rslbliss/r_logistic_ws .

Addtional Information

Figure 1 - Total number of crosses among the progeny type and the initial male

```
##   DATA BANK

coitus_freq <- data.frame(progeny=as.factor(c("Male 1","Male 1&2","Male 2")),

group=(c("A","A","A","B","B","B","C","C","C","A","A","A","B","B","B","C","C",
"C")),

crosses = c(41,0,0,18,14,3,1,11,32,37,1,0,15,16,4,1,5,28),

male_order=c((rep("Transgenic",9)),(rep("Wild-type",9))))
```

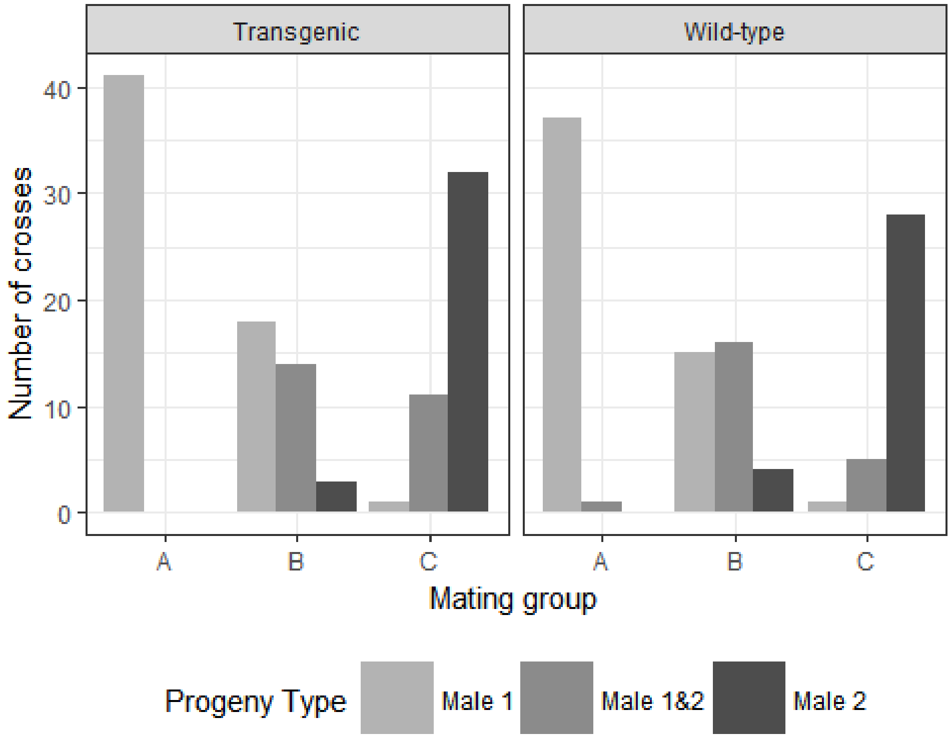

Supplement: S2 File — Detailed statistical analysis conducted for each experiment and extra information. (PDF) [file pone.0193164.s002.pdf]
